# Long-Term Responses of Mediterranean Mountain Forests to Climate Change, Fire and Human Activities in the Northern Apennines (Italy)

**DOI:** 10.1007/s10021-020-00587-4

**Published:** 2020-12-02

**Authors:** César Morales-Molino, Marianne Steffen, Stéphanie Samartin, Jaqueline F. N. van Leeuwen, Daniel Hürlimann, Elisa Vescovi, Willy Tinner

**Affiliations:** 1 Institute of Plant Sciences and Oeschger Centre for Climate Change Research, University of Bern, Altenbergrain 21, CH-3013 Bern, Switzerland; 2 Swiss Federal Institute for Forest, Snow and Landscape Research WSL, A Ramél 18, CH-6593 Cadenazzo, Switzerland

**Keywords:** *Abies alba*, Climate change, *Fagus sylvatica*, Holocene, Fire ecology, Land-use, Late glacial, Legacy effects, Paleoecology, Pollen analysis

## Abstract

*Fagus sylvatica* (beech) dominates the montane forests of the Apennines and builds old-growth high-conservation value stands. However, recent severe drought-induced diebacks raise concern on the future persistence of these forests and of Southern European mesophilous woodlands overall, growing at their dry edge. To explore the history of Apennine beech-dominated forests, we draw on the multiproxy paleoecological record from Lago Verdarolo, which includes a robust vegetation-independent temperature reconstruction. Numerical techniques are used to investigate the drivers of long-term Mediterranean mountain forest dynamics. Specifically, we focus on disentangling the ecological factors that caused the shift from high-diversity mixed forests to beech-dominated stands and on assessing the occurrence of legacy effects on present-day forests. Abrupt climate change largely drove vegetation dynamics during the Late Glacial and Early Holocene. Species-rich mixed *Abies alba* (silver fir) forests dominated about 10,500—5500 years ago, under rather dry and warmer-than-today conditions (+ 1—2 °C) and limited fire occurrence. Cooler and moister summers and increasing fire activity caused declines in several fire-sensitive temperate deciduous trees (for example, *Ulmus, Tilia, Fraxinus*) and favored the establishment of fir-beech forests around 5500 years ago. Further enhancement of fire activity and farming around 2000 years ago led to local *Abies alba* extinction and forest impoverishment. We conclude that the currently widespread monospecific Apennine beech forests are the result of multi-millennial land-use intensification superimposed on Late Holocene cooling and moistening. Given their higher drought-tolerance compared to beech stands, reviving ancient species-rich mixed fir forests represents a feasible and ‘tested’ possibility to adapt forests to climate change.

**Electronic supplementary material:**

The online version of this article (10.1007/s10021-020-00587-4) contains supplementary material, which is available to authorized users.

## Highlights

- Mixed Abies-dominated forests thrived under mid-Holocene warmer-than-present climate.
- Extant Apennine monospecific Fagus forest dominance was driven by historical land-use.
- Reviving diverse mid-Holocene forests may help overcome predicted Fagus diebacks.

## Introduction

Global change, including human-induced climatic change and rapid shifts in land-use, is posing serious threats to forest ecosystems and diversity and urging the adoption of adaptation and mitigation measures across Europe (Thuiller and others 2005; García-Valdés and others 2015; Ruiz-Benito and others 2017). Climate change impacts are predicted to be particularly severe on stands dominated by temperate mesophilous and boreal tree species in the Mediterranean peninsulas, at the dry edge of their distribution ranges (Piovesan and others 2008; Galiano and others 2010; Gazol and others 2015). In this context, the Apennines are home to highly diverse forests (Vacchiano and others 2017) dominated by many European temperate and boreal tree species that reach here the southern limit of their distribution ranges (San-Miguel-Ayanz and others 2016). Currently, such species are experiencing growth decreases associated to ongoing climate change (Piovesan and others 2008; Gazol and others 2015). *Fagus sylvatica* (beech) is a key species (> 600,000 ha) of present-day Apennine mid to high-elevation forests ($\sim$ 800–1800 m asl), including some old-growth stands of particularly high conservation value (Sabatini and others 2018). However, dendroecological research has shown that southern beech populations have exhibited severe growth reductions and symptoms of decline under recent drought, which suggest that their future persistence under forecasted warmer and drier summers is uncertain (Piovesan and others 2008; Dorado-Liñán and others 2019). Additionally, although some valuable contributions on the disturbance ecology of Apennine forests exist (van Gils and others 2010; Vescovi and others 2010a), this topic, which is crucial for ecosystem functioning and future management, remains largely unknown, especially in the long term (Vacchiano and others 2017).

Paleoecology’s long-term perspective enables to explore ecosystem dynamics under different climatic scenarios and disturbance regimes and therefore to get relevant insights into the aforementioned questions. For instance, regional paleoecological records have shown that monospecific beech forests established in relatively recent times following the decline of *Abies* and other mesophilous deciduous trees (for example, Watson 1996; Vescovi and others 2010a; Branch 2013). Further, recent interdisciplinary studies combining paleoclimatic, paleoecological and dynamic modeling efforts have suggested an ecologically unexpected potential of mixed *Abies alba* (silver fir) forests to cope with conditions significantly warmer and drier than today’s (Tinner and others 2013). Despite these progresses, major relevant gaps in the ecological knowledge of the Apennine forests persist, particularly the causes of past paramount vegetational shift such as the late Holocene expansion of *Fagus* and its ecological consequences. Several hypotheses have been formulated to explain the mass expansion of beech forests, namely climate change, human impact (and the use of fire), or a combination of both (Watson 1996; Vescovi and others 2010a; Branch 2013). However, the lack of robust independent paleoclimatic reconstructions, the usually insecure chronologies (most of the available records are bulk dated; see Finsinger and others 2019), the paucity of charcoal records, and the relatively low taxonomical resolution of pollen records have so far hindered comprehensive and quantitative assessments of the responses of the main forest trees of the Apennines to past climate change and fire disturbance. Similarly, the potential occurrence of legacy effects in the current composition and structure of Apennine forests have not been fully addressed yet (but see Tinner and others 2013; Branch and Marini 2014).

Recently published quantitative Holocene summer temperature reconstructions inferred from fossil chironomid (non-biting midges) assemblages from two lakes in the Northern Apennines (Samartin and others 2017) open the door to more thorough and independent assessments of the main drivers of Mediterranean mountain forest dynamics. In this paper, we present novel well-dated multi-proxy paleoecological data (pollen, spores, macrofossils, microscopic and macroscopic charcoal) from Lago Verdarolo (Tuscan-Emilian Apennines). These data are compared with an independent Holocene temperature reconstruction from the same lake, which was replicated in a second site in the Apennines (Samartin and others 2017), using multivariate and regression techniques to provide new insights into the long-term responses of Apennine forests to climate change, fire activity and human disturbance. Our specific aims are: (1) to track the vegetation responses to Late Glacial and Early Holocene abrupt climatic changes, (2) to assess the long-term ecology of Early and Mid-Holocene mixed forests, (3) to disentangle the ecological factors driving the shift from highly diverse mixed forests to beech-dominated stands, and (4) to assess the occurrence of legacy effects on present-day forests. In a final step, we aim at providing recommendations for the future management of these forests under future warmer and drier climate.

## Material and Methods

### Study Site

**Figure 1:**
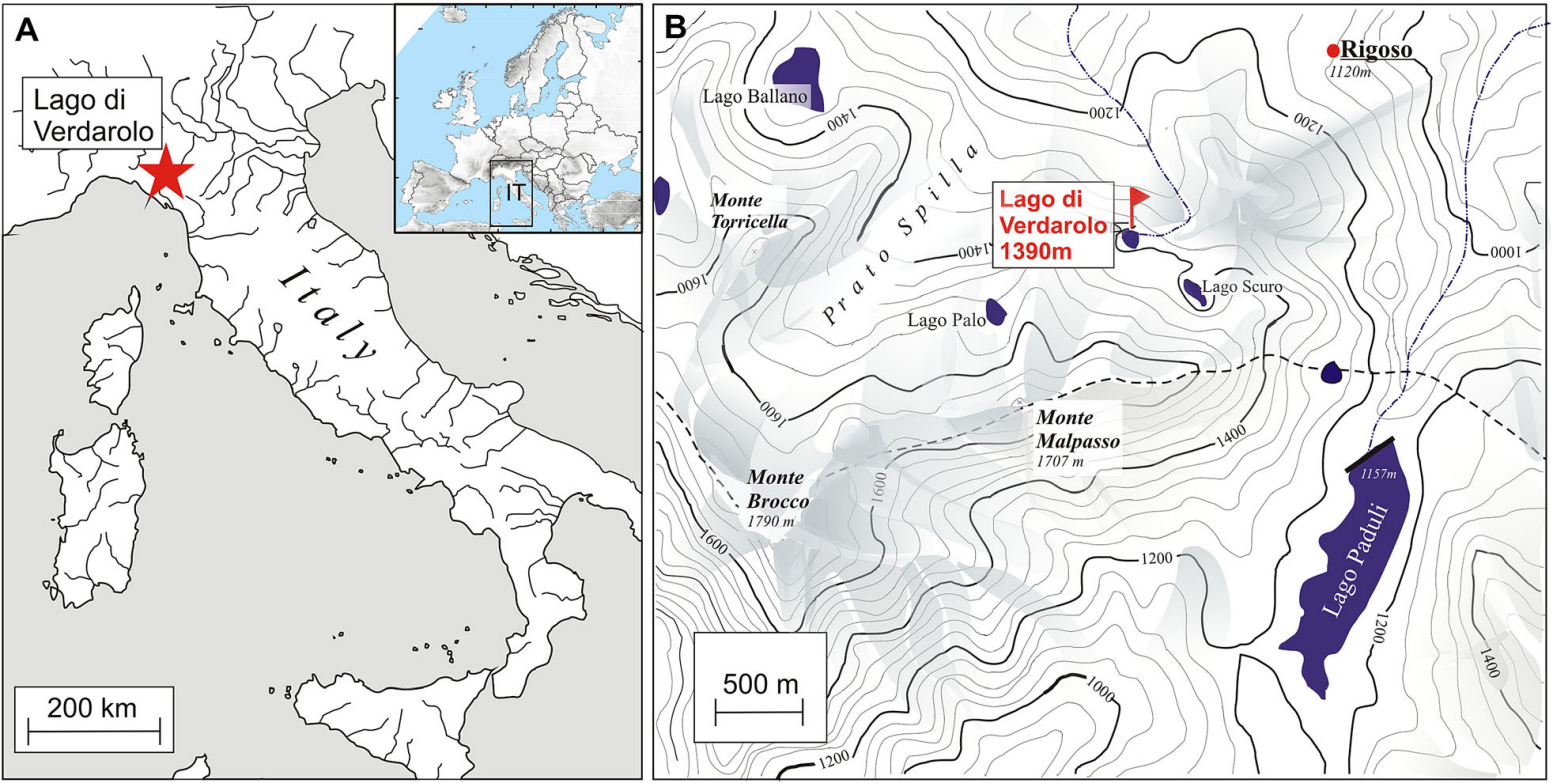
** A** Location of Lago Verdarolo in the Northern Apennines (northern Italy). The inset map shows the location of Italy (IT) in Europe. **B** Topographic map of the Tuscan-Emilian Apennines with the location of relevant paleoecological sites: Lago Verdarolo (this paper; Samartin and others 2017) and Prato Spilla area (five sites, Prato Spilla A–E; Lowe 1992; Ponel and Lowe 1992).

Lago Verdarolo (44°21′33.2″N, 010°07′23.2″E, 1390 m asl) is a small ($\sim$ 1 ha) and shallow (maximum depth $\sim$ 3 m) glacial lake located in the protected area *‘Parco dei Cento Laghi’* in the Northern Apennines (Italy; Figure 1). Today, the lake has no inlet and only one small outlet on the north shore. The dominant bedrocks in the catchment are sandstone and limestone. Between 1905 and 1960 CE, the lake was dammed and used as water reservoir for a hydropower plant. Today’s climate at Lago Verdarolo is cool temperate with a mean annual temperature (*T*) of about 6 °C (*T*_January_ = − 1.5 °C, *T*_July_ = 15 °C), mean annual precipitation of $\mathrm{about}$ 2500 mm, and no summer drought (*P*_summer_ = 350 mm) despite its location in the Mediterranean Basin. Modern vegetation around the lake consists of young, closed and almost monospecific oromediterranean beech forests. In the study area, submediterranean forests are widespread up to around 800—1000 m asl and are dominated by *Quercus pubescens, Q. cerris, Sorbus torminalis, Acer monspessulanum, A. campestre, Fraxinus ornus, Ostrya carpinifolia* and *Castanea sativa* (locally dominant on favorable habitats).

### Coring and Chronology

In August 2009, we retrieved two parallel cores from the deepest section of Lago Verdarolo with a modified Livingstone piston corer. The two cores were correlated according to their lithostratigraphy to produce a master sequence (855-cm long). The chronology relies on 13 accelerator mass spectrometry (AMS) radiocarbon dates of terrestrial plant macrofossils (Table S1), which were calibrated to calendar years Before Present (cal BP, with 0 BP = 1950 CE) using the IntCal13 calibration curve (Reimer and others 2013). The depth-age relationship was then modeled using generalized mixed-effect regression within the framework of general additive modeling (GAM; Heegard and others 2005). Given the unrealistic estimates provided by this model for the topmost section of the sequence, we used linear interpolation to estimate the ages of the samples between the uppermost radiocarbon date and the core top. The radiocarbon dates and the age-depth model were first published in Samartin and others (2017).

### Pollen, Charcoal, and Macrofossil Analyses

In the laboratory, we prepared 81 sediment sub-samples of 1 cm^3^ for pollen analysis following standard protocols (Moore and others 1991), but sieving at 500 µm to allow for large pollen grains, charcoal and other palynomorphs to be represented. Tablets with a known amount of *Lycopodium* spores were added at the beginning of the treatment to estimate pollen concentrations (Stockmarr 1971). Pollen influxes were calculated dividing pollen concentrations by the sediment deposition time (yr cm^−1^). We identified pollen grains using determination keys and photographic atlases (for example, Moore and others 1991; Reille 1992; Beug 2004) and the reference collection of the *Institute of Plant Sciences* at the University of Bern, up to a minimum terrestrial pollen sum of 400 grains, which excludes pollen from aquatic plants as well as fern spores. We did not distinguish *Quercus pubescens*-t. (t. = type) and *Q. cerris*-t. during pollen analysis, so we have grouped both into ‘deciduous *Quercus*’. In the pollen diagrams, we have represented pollen and spore percentages calculated with respect to the above defined terrestrial pollen sum. In the same slides used for pollen analysis, we quantified charcoal particles between 10 and 500 µm (referred to as ‘microscopic charcoal’ hereafter) according to Tinner and Hu (2003) and Finsinger and Tinner (2005). We estimated charcoal concentrations and influxes using the same approach as for pollen. Macrofossil analysis was conducted on 22 sediment sub-samples of 10 cm^3^ sieved through a mesh of 200 µm. We then identified macrofossils using a stereomicroscope (10–50 ×), macrofossil atlases and identification keys (for example, Schoch and others 1988; Tomlinson 1995; Lévesque 1998; Birks 2013), and the reference collection of the *Institute of Plant Sciences* at the University of Bern. In the macrofossil diagrams, the results are expressed as concentrations of macrofossils per 10 cm^3^ of sediment. We also counted charcoal particles (> 200 µm; called ‘macroscopic charcoal’ hereafter) during macrofossil analyses, calculating influxes (# cm^−2^ y^−1^) dividing by the volume of sediment sieved (cm^3^) and the sediment deposition time (y cm^−1^).

### Numerical Analyses

We delimited local pollen assemblage zones (LPAZ) using the optimal splitting by sums-of-squares technique (Birks and Gordon 1985), determining the number of statistically significant LPAZs by comparison with the broken-stick model (Bennett 1996).

To investigate the occurrence of underlying environmental gradients in the pollen data and to quantify vegetation responses to Holocene climate variability and fire activity we conducted unconstrained and constrained ordination analyses (Legendre and Birks 2012) in Canoco 5 (ter Braak and Šmilauer 2012). We first ran a detrended correspondence analysis (DCA) on square-root transformed percentage pollen data, detrending by segments, and without down-weighting rare species to check whether ordination techniques based on linear or unimodal response models were more appropriate (Legendre and Birks 2012). As the length-of-gradient of Axis 1 was rather short (1.62 standard deviation units of turnover), we decided to use methods based on linear response models like principal component analysis (PCA) and redundancy analysis (RDA) for further analysis (Legendre and Birks 2012; Šmilauer and Lepš 2014). For the unconstrained ordination analyses, that is, DCA and PCA, we used the entire Lago Verdarolo pollen dataset (*n* = 81 samples).

In a second step, we carried out RDA to get quantitative insights into the response modes of vegetation (inferred from percentage pollen data) to summer (July) air temperature anomalies (in °C, quantitatively reconstructed from chironomid assemblages and calculated with respect to the mean pre-industrial late Holocene, that is, 2000–100 cal BP, *T*_July_; Samartin and others 2017) and fire activity (inferred from microscopic charcoal influxes, in # cm^−2^ y^−1^). We tested the statistical significance of the relationships observed between the response variables (pollen types) and the environmental variables (*T*_July_ anomaly, microscopic charcoal influx) using Monte Carlo permutation tests (999 iterations, reduced model, unrestricted permutations; Šmilauer and Lepš 2014). Further, we used variation partitioning to quantify the independent and shared amount of variation in the pollen dataset explained by each of the environmental variables. Finally, we modeled the responses of major tree and shrub taxa of the Lago Verdarolo paleoecological record such as *Abies*, *Fagus*, *Fraxinus excelsior*-t., *Tilia*, *Ulmus* and *Corylus* (pollen abundance: %) to summer air temperature (*T*_July_ anomaly: °C) and fire activity (microscopic charcoal influx: # cm^−2^ y^−1^) fitting generalized additive models (GAM; Hastie and Tibshirani 1990; Colombaroli and others 2010; Šmilauer and Lepš 2014). We assumed a Poisson distribution for the response variables, used a log link function, limited the polynomial order of the fitted function (that is, degrees of freedom; *DF*) to two, and finally chose the most parsimonious model improving the null model (not incorporating the environmental variables) using stepwise selection based on the Akaike Information Criterion corrected for small sample size (*AICc*; Šmilauer and Lepš 2014). The performance of the models was evaluated using their *R*^2^ (%) and Monte Carlo permutations (Šmilauer and Lepš 2014). For RDA and response curves we used a reduced dataset consisting of those samples with pollen, *T*_July_ and microscopic charcoal data available simultaneously (*n* = 35 samples). Response curves were fitted in Canoco 5 (ter Braak and Šmilauer 2012).

## Results and Interpretation

### Vegetation and Fire History

**Figure 2:**
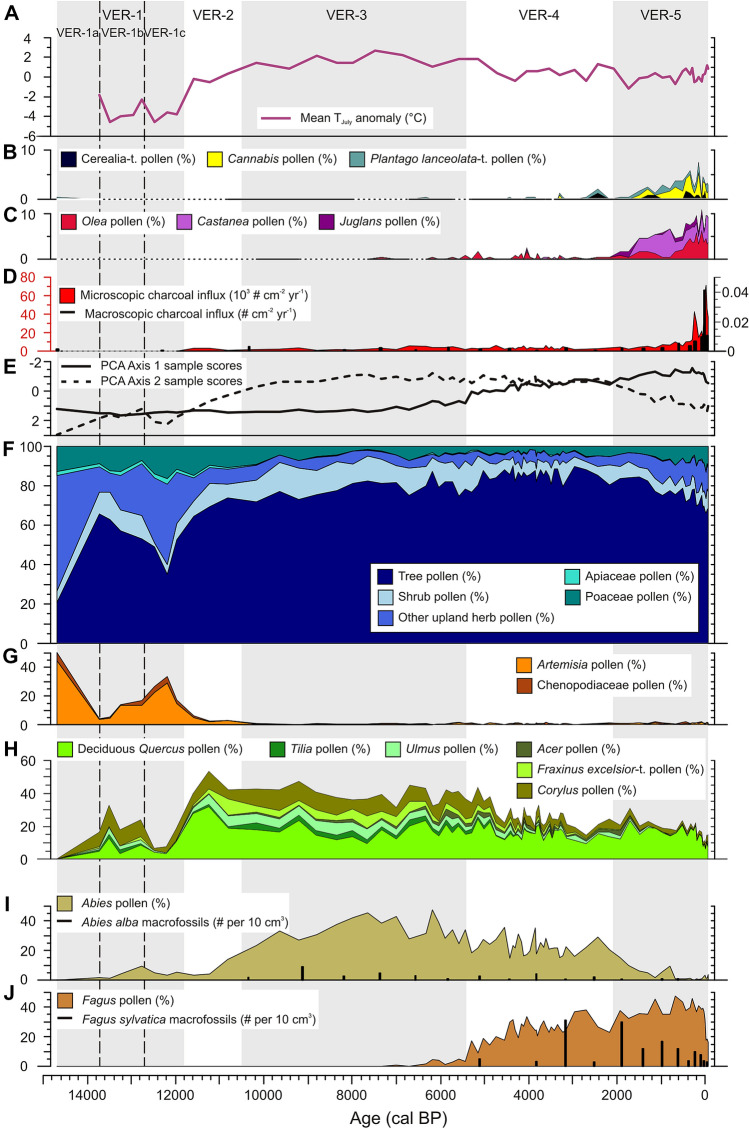
Paleoecological record from Lago Verdarolo (northern Italy): vegetation dynamics and their drivers. **A** Chironomid-based reconstruction of mean July air temperature anomalies (°C) with respect to the mean of pre-industrial late Holocene (2000–100 cal BP = 50 BCE-1850 CE; Samartin and others 2017). **B**, **C** Relative abundances (%) of the most important herb (Cerealia-t., *Cannabis*, *Plantago lanceolata*-t.) and tree (*Olea*, *Castanea*, *Juglans*) pollen indicators of farming activities (t. = type). **D** Microscopic (10–500 µm; pollen slides) and macroscopic (> 200 µm; sieved sediment) charcoal influx values (# cm^−2^ y^−1^), proxies for local to regional fire activity. **E** Principal component analysis (PCA) Axis 1 and 2 sample scores. **F** Temporal changes in vegetation structure (openness) based on the relative abundances (%) of tree, shrub and upland herb (including Poaceae and Apiaceae) pollen. **G**, **H** Relative abundances (%) of pollen from taxa typical of steppic environments (*Artemisia*, Chenopodiaceae) and of mixed deciduous forests (deciduous *Quercus*, *Tilia*, *Ulmus*, *Acer*, *Fraxinus excelsior*-t., *Corylus*). **I**, **J** Pollen (curves; %) and macrofossil (histograms; # per 10 cm^3^) abundances of *Abies* and *Fagus*, dominant trees of the regional montane forests. Grey and white bands are delimiting the five statistically significant local pollen assemblage zones (LPAZ) identified in the pollen record (VER-1 to VER-5), and the dashed lines are separating the three sub-zones within the LPAZ VER-1.

The paleoecological record of Lago Verdarolo starts around 14,700 cal BP, although this age must be taken with caution because the age-depth model relies on extrapolation for the basal section of the sedimentary sequence (Figure S1). The oldest statistically significant LPAZ, VER-1 ($\sim$ 14,700—11,700 cal BP) can be sub-divided into three sub-zones: VER-1a to VER-1c. At the beginning of VER-1a ($\sim$ 14,700—13,700 cal BP), non-arboreal (herb) pollen (NAP) dominates ($\sim$ 70%), particularly *Artemisia*, Poaceae and Chenopodiaceae, whereas arboreal (tree and shrub) pollen (AP) percentages are very low ($\sim$ 30%), mostly *Pinus sylvestris*-t. and *Juniperus*-t. (Figures 2, S2). This points to a regional vegetation dominated by cold steppe with scattered pioneer trees and shrubs. Find of few pollen grains of *Abies* and *Picea* suggest their regional persistence at lower elevations (Figure S2). Later during this sub-zone, herb pollen decreases dramatically (particularly steppics such as *Artemisia* and Chenopodiaceae), to be mostly replaced with *Pinus sylvestris*-t. (up to 50%) and *Corylus* (up to 10%). This suggests an ascent of the tree line, most likely below the site, given that AP below 80% points to open vegetation (Figure S3). During VER-1b ($\sim$ 13,700—12,650 cal BP), AP decreases. Despite this general decline, mostly related to *Pinus sylvestris*-t., the moderate increases of *Abies* and deciduous trees and shrubs (deciduous *Quercus*, *Tilia*, *Ulmus*, *Corylus*) suggest a regional spread of temperate woodlands (Figures 2, S2). Since about 13,500 cal BP, NAP increase discreetly (notably *Artemisia* and Chenopodiaceae), continuing the re-expansion of steppic environments that peaked during VER-1c ($\sim$ 12,650—11,750 cal BP; Figures 2, S2). Meanwhile, AP drops (minimum of $\sim$ 35%), mostly driven by declines of temperate deciduous trees and shrubs (for example, deciduous *Quercus*, *Tilia*, *Ulmus*, *Corylus*), which subsequently begin to recover at the end of VER-1c. Findings of macroscopic charcoal fragments suggest the occurrence of fire in the catchment.

The abrupt increase in deciduous *Quercus* and the marked rises in *Ulmus*, *Fraxinus excelsior*-t., *Betula* and *Corylus* pollen percentages indicate that the rapid spread of mixed deciduous forests at the expense of steppic vegetation and pine woods continued during VER-2 ($\sim$ 11,750—10,500 cal BP; Figures 2, S2). *Abies* pollen percentages start to increase at about 11,000 cal BP, paralleling decreasing abundances of deciduous trees such as *Quercus* and *Betula*. Microscopic charcoal influx values are very low throughout this zone (Figure 2). Consistently high AP percentages (> 85%) during VER-3 ($\sim$ 10,500—5400 cal BP) indicate that closed forests dominated for several millennia during the Early and Mid-Holocene around Lago Verdarolo (Figure 2). The remarkable abundance of *Abies* in the pollen assemblages (up to 50%) and its continuous macrofossil record suggest that fir was particularly abundant in the local forests, likely mixed with deciduous *Quercus*, *Ulmus*, *Fraxinus excelsior*-t., *Tilia*, *Acer* and *Corylus* (Figures 2, S2–S3). In contrast, pioneer or boreal trees such as *Betula* and *Pinus sylvestris*-t. reach very low pollen representation that will continue until present (Figure S2). At about 6700 cal BP, a moderate decrease in *Abies* pollen percentages is recorded, synchronous with notable increases of deciduous *Quercus*, *Corylus* and Poaceae, slightly rising microscopic charcoal influxes and the occurrence of scattered *Plantago lanceolata*-t. pollen grains (Figure 2). This suggests moderate opening of the forest and a shift in vegetation composition toward higher representation of disturbance-tolerant species, probably as a consequence of Neolithic use of fire for farming purposes. The latter increase in *Abies* pollen percentages indicates that fir recovered from this disturbance episode before undergoing a more pronounced and lasting decline around 6000 cal BP, coupled with a spread of *Corylus* and *Pteridium aquilinum*, which was again related to fire disturbance (moderate charcoal influx maximum; Figures 2, S2). The first pollen grains of *Fagus* date to about 7000 cal BP and beech pollen occurs regularly after about 6500 cal BP (Figure 2). However, its low pollen representation (< 5%) alongside the lack of macrofossil finds during VER-3 suggest that beech was present in the region but without major relevance (either locally or regionally).

The steady and prominent increase in *Fagus* pollen abundance is the main feature of VER-4 ($\sim$ 5400—2050 cal BP), together with overall lower frequencies of *Abies* and most of the deciduous trees and shrubs (for example, *Fraxinus excelsior*-t., *Tilia*, *Ulmus*, *Corylus*) that co-dominated previously (VER-3; Figure 2). Pollen data suggest that beech rapidly spread around Lago Verdarolo from around 5400 cal BP to form mixed beech-fir forests that replaced the previously dominant mixed fir forests. The macrofossil record supports the occurrence of *Abies alba*-*Fagus sylvatica* forests in the Lago Verdarolo catchment (Figure 2). Although not so conspicuously as *Fagus*, *Carpinus betulus*-t. also expanded regionally since about 5200 cal BP, whereas the first occurrences of the submediterranean trees *Fraxinus ornus*-t. and *Ostrya*-t. (moderate spread at $\sim$ 3500 cal BP) also date to this period (Figure S2). Concerning anthropogenic pollen indicators, the continuous curve of *Plantago lanceolata*-t. since the beginning of this LPAZ suggests regional farming (Figure 2). At about 2500 cal BP, *Abies* pollen percentages start to drop, microscopic charcoal concentrations and influxes increase slightly, the first Cerealia-t. pollen grains are recorded, and *Corylus* percentages increase (Figures 2, S2). These data suggest that arable and pastoral farming involving the use of fire (moderate charcoal maximum at $\sim$ 2000 cal BP) probably triggered the regional and local decline of fir around 2000 cal BP.

Decreasing, although still high, AP percentages coupled with increasingly abundant NAP characterize VER-5 ($\sim$ 2050 cal BP—today), which indicates the opening of clearings in a rather forested landscape or the establishment of wooded pastures (Figure 2). Among trees, fir continued declining while beech kept expanding, although less pronouncedly. Indeed, there is no *Abies alba* macrofossil since about 1000 cal BP and *Abies* pollen percentages collapsed completely at about 1200 and about 600—400 cal BP (Figure 2). Other trees that were important in the mixed forests of the Early and Mid-Holocene such as *Acer*, *Carpinus betulus*-t., *Fraxinus excelsior*-t., *Tilia* and *Ulmus* also have discontinuous records or are completely absent in the uppermost pollen assemblages. The abundances of anthropogenic pollen indicators such as Cerealia-t., *Cannabis*, *Plantago lanceolata*-t., *Castanea*, *Juglans* and *Olea* increase notably during the past two millennia, particularly after about 1500 cal BP (Figure 2). Fire activity has also followed an increasing trend during the past 2000 years, particularly during the past 400 years according to microscopic and macroscopic charcoal influxes (Figure 2). One might speculate that the observed increase in charcoal influx is an artifact related to the uncertainties of the age-depth model. However, the correspondence of a distinct peak in macroscopic charcoal influx with a documented recent local fire event ($\sim$ 30 years ago) supports the robustness of the results.

### Ordination and Species Response Curves

**Figure 3:**
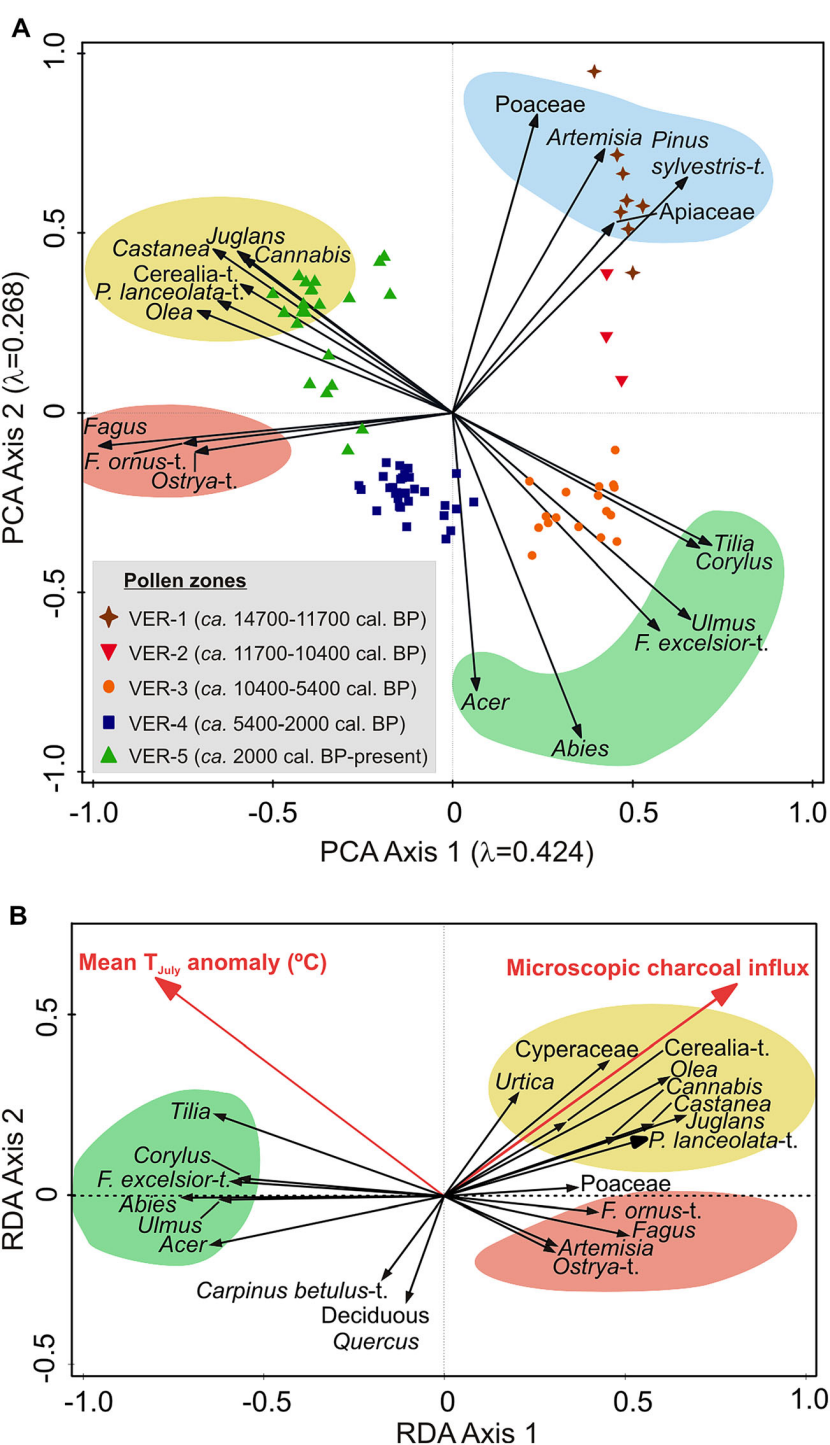
Ordination analyses on the Lago Verdarolo paleoecological record. **A** Principal component analysis (PCA) on the entire pollen dataset (square-root transformed percentages), that is, Late Glacial and Holocene (*n* = 81 samples). The first and second axes explain 42.4% and 26.8% of the variation in the pollen dataset, respectively. **B** Redundancy analysis (RDA) on a reduced dataset including samples with pollen, chironomid-inferred mean July air temperature and microscopic charcoal data simultaneously (*n* = 35 samples). Response variables are the pollen taxa (square-root transformed pollen percentages), while the environmental variables are chironomid-inferred mean July air temperature anomalies (°C) with respect to the mean of the period 2000–100 cal BP (Samartin and others 2017) and microscopic charcoal influx (# cm^−2^ y^−1^). The environmental variables explain together the 27.9% of the variation in the Holocene pollen dataset (microscopic charcoal influx = 10.8%, Mean *T*_July_ anomaly = 10.3%, Shared = 6.9%). Abbreviations: *F. excelsior*-t. = *Fraxinus excelsior*-t., *F. ornus*-t. = *Fraxinus ornus*-t., *P. lanceolata*-t. = *Plantago lanceolata*-t.

The first two PCA axes explain together 69.2% of the variation in the Lago Verdarolo pollen dataset (Axis 1 42.4%, Axis 2 26.8%; Figure 3A). Positive species scores in Axis 1 are reached by, on the one hand, taxa typical of steppic or open vegetation (for example, *Artemisia*, Poaceae, Apiaceae) and the pioneer *Pinus sylvestris*-t. and, on the other hand, meso-thermophilous trees and shrubs (for example, *Abies, Acer, Corylus, Fraxinus excelsior*-t., *Tilia* and *Ulmus*). These taxa are dominant in Late Glacial and Early and Mid-Holocene pollen assemblages, which also feature high sample scores. In contrast, Axis 1 sample scores of Late Holocene pollen assemblages as well as species scores of their dominant/most characteristic taxa, that is, *Fagus*, anthropogenic pollen indicators (for example, Cerealia-t., *Plantago lanceolata*-t., *Cannabis*, *Castanea*, *Olea*), and submediterranean trees (that is, *Ostrya*-t., *Fraxinus ornus*-t.) are negative (Figure 3A). These results suggest that the first axis of the PCA is reflecting a gradient in anthropogenic disturbance, with positive values associated with less disturbed vegetation and negative values linked to increasing human-induced disturbance (mostly farming) and beech forests. Indeed, when plotting PCA Axis 1 sample scores against time (Figure 2E) it becomes evident that the PCA curve mostly runs parallel to that of *Fagus* (after its establishment; Figure 2J) and to those of anthropogenic pollen indicators (Figure 2B, C). On PCA Axis 2, strongly positive species scores correspond to steppic taxa particularly abundant in Late Glacial pollen assemblages (for example, *Artemisia*, Poaceae, *Pinus sylvestris*-t.), taxa typical of the Early and Mid-Holocene mixed meso-thermophilous forests (for example, *Abies*, *Acer*, *Fraxinus excelsior*-t.) bear strongly negative scores, and taxa relevant in Late Holocene pollen assemblages like *Fagus*, anthropogenic pollen indicators (for example, Cerealia-t., *Plantago lanceolata*-t., *Cannabis*, *Castanea*) and submediterranean trees have intermediate sample scores, from slightly negative to moderately positive (Figure 3A). Plotting PCA Axis 2 sample scores against age shows that the curve follows very similar trends to those of tree pollen, thus suggesting a close connection with vegetation openness (Figure 2E, F).

Microscopic charcoal influx and chironomid-inferred mean *T*_July_ anomalies explain together 27.9% of the variation in the Holocene pollen dataset (*P* = 0.001). Separately, microscopic charcoal influx and mean *T*_July_ anomalies account for 10.8% (*P* = 0.002) and 10.3% (*P* = 0.004) of that variation, respectively, while the shared variation explained is 6.9%. Crops (for example, Cerealia-t., *Cannabis*, *Olea*, *Castanea*) and other taxa related to farming activities (for example, *Plantago lanceolata*-t., *Urtica*) show a highly positive correlation with microscopic charcoal influx (Figure 3B). Poaceae, *Artemisia*, *Ostrya*-t., *Fraxinus ornus*-t. and *Fagus* are positively correlated with microscopic charcoal influx (Figure 3B). In contrast, strongly negative correlation exists between microscopic charcoal influx and many meso-thermophilous deciduous trees (for example, *Acer, Carpinus betulus*-t.*,* deciduous *Quercus*, *Ulmus, Fraxinus excelsior*-t., *Tilia*) and *Abies* (Figure 3B). Regarding mean T_July_ anomalies, most components of the Early and Mid-Holocene forests at Lago Verdarolo (*Abies*, *Tilia*, *Fraxinus excelsior*-t., *Ulmus*, *Corylus*, *Acer*) are quite positively correlated, whereas *Fagus*, *Artemisia*, Poaceae, *Fraxinus ornus*-t. and *Ostrya*-t. show strongly negative correlations (Figure 3B).

**Figure 4:**
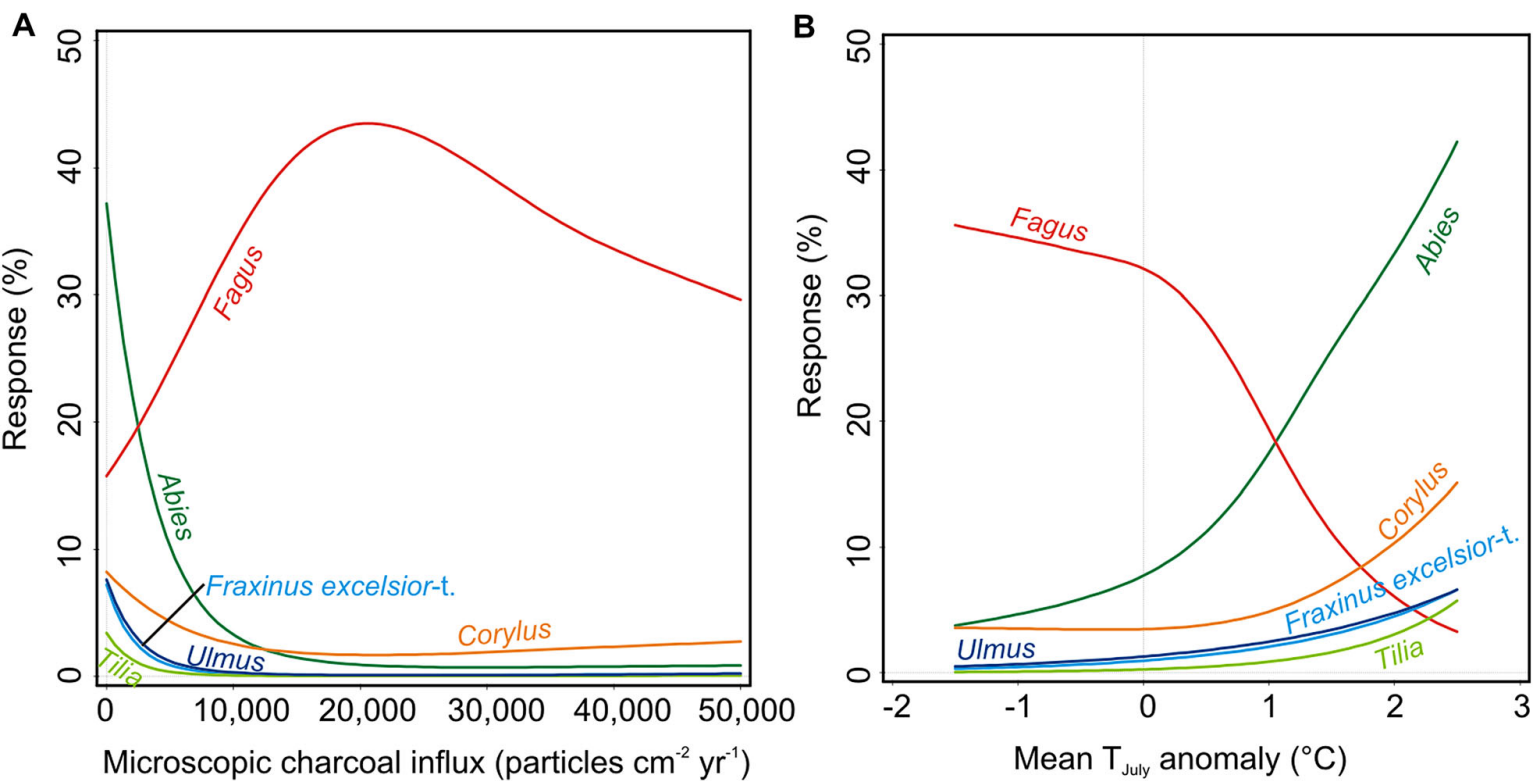
Response curves of the main trees and shrubs in the pollen record from Lago Verdarolo to **A** fire occurrence (inferred from microscopic charcoal influx) and **B** mean July air temperature anomalies with respect to the mean of the period 2000–100 cal BP (inferred from chironomid assemblages; Samartin and others 2017) fitted using general additive models (GAM).

The response curves (GAMs) of *Abies*, *Ulmus*, *Fraxinus excelsior*-t. and *Tilia* to microscopic charcoal influx provide compelling evidence about the sensitivity of these trees to increasing fire activity (Figures 4A, S4A; Table 1). Our data also suggest that *Corylus* responded negatively to light or moderate fire activity, whereas it was favored by high regional burning (Figures 4A, S4A; Table 1). In contrast, light to moderate fires likely boosted *Fagus* but high fire activity affected it negatively (Figures 4A, S4A; but note the moderate robustness of the model, Table 1). The GAMs provide firm evidence on the positive responses of *Abies*, *Corylus*, *Fraxinus excelsior*-t., *Ulmus* and *Tilia* to markedly warmer summers (up to $\sim$ 2.5 °C higher than the average of the past two millennia; Figures 4B, S4B; Table 1). In clear contrast, the highest abundances of *Fagus* occurred under cooler (and wetter) summers (Figures 4B, S4B; Table 1).

**Table 1:** Results of the General Additive Models (GAM) Fitted to Model the Responses of the Main Trees and Shrubs in the Lago Verdarolo Pollen Record to Fire Occurrence (Inferred From Microscopic Charcoal Influxes, Particles cm^−2^ y^−1^) and Mean July Air Temperature Anomalies (°C) with Respect to the Mean of the Period 2000–100 cal BP (Samartin and others 2017).

	Model deviance	*DF* model	*AICc*	*R*^2^ (%)	*F*	*P*
Microscopic charcoal influx						
*Abies*	176.16	2	312.71	60.9	142.2	< 0.00001
*Fagus*	420.37	2	572.36	23.1	63.0	< 0.00001
*Fraxinus excelsior*-t	33.006	2	100.62	59.8	24.3	< 0.00001
*Tilia*	27.391	2	69.63	45.7	11.7	0.00016
*Ulmus*	30.409	2	104.83	63.4	25.9	< 0.00001
*Corylus*	38.63	2	156.73	46.8	17.0	< 0.00001
Mean *T*_July_ anomaly						
*Abies*	277.16	2	413.76	38.5	88.5	< 0.00001
*Fagus*	374.57	2	526.48	31.5	87.3	< 0.00001
*Fraxinus excelsior*-t	59.459	1	124.62	27.6	22.7	0.00004
*Tilia*	25.963	1	65.88	48.5	24.4	0.00002
*Ulmus*	63.515	1	135.45	23.6	19.7	0.0001
*Corylus*	44.366	2	162.50	38.9	14.0	0.00004

*t*. = type.

## Discussion

### Vegetation Responses to Late-Glacial and Early Holocene Climate Change

The initial replacement of steppe-tundra or alpine meadows with wooded vegetation (mostly pinewoods and *Corylus* thickets) around Lago Verdarolo from around 14,700 to 13,700 cal BP (Figures 2, S2) was most likely a response to the rapid and abrupt warming at the onset of the Bølling/Allerød interstadial (+ 2.5—3 °C according to chironomid-based temperature reconstructions from the neighboring southern Alps ($\sim$ 250 km away; Samartin and others 2012a, b). Unfortunately, the coarse resolution of the pollen record, the insecure chronology (based on extrapolation) and the unavailability of local temperature reconstruction for this period hinder exploring in more detail this marked vegetational shift (Figures 2, S1-S2). Progressively warmer summers (+ 0.5 °C; Samartin and others 2017) and wetter conditions (Magny and others 2006) during the remainder of the Bølling/Allerød interstadial ($\sim$ 13,700—12,650 cal BP) drove the regional spread of mixed deciduous woodlands (deciduous *Quercus*, *Tilia*, *Ulmus*, *Corylus*) with *Abies* at the expense of pinewoods (Figure 2). According to the macrofossils found (needles can be determined to species level), the available fossil evidence from the Italian Peninsula (including macrofossils and wood; Tinner and others 2013), and the current distribution and ecology of European *Abies* species (Caudullo and Tinner 2016), it is certain that *Abies alba* was the fir species present around Lago Verdarolo during the Late Glacial and the Holocene. Similar vegetation dynamics occurred during the Late Glacial interstadial in other areas of the Northern Apennines, as shown by the paleoecological records from Prato Spilla (Lowe 1992, but see discussion about the chronology in Vescovi and others 2010a) and Lago del Greppo (Vescovi and others 2010a). At Lago del Greppo, direct radiocarbon-dating of *Abies alba* macrofossils provided firm evidence on the local presence of fir above 1400 m asl at about 13,000 cal BP (Vescovi and others 2010a). This early spread of fir and temperate broadleaved trees relates to the prominent role played by the Apennines as glacial refugia (Tzedakis and others 2013; Guido and others 2020). The transient minor reduction of temperate trees alongside certain spread of steppic taxa at 13,500 cal BP could have been due to cooler conditions (− 1 °C) at about 13,700—13,500 cal BP (Figure 2; Samartin and others 2017), also registered on the Southern Alps (Samartin and others 2012a, b). Significant cooling (− 2—2.5 °C) during the Younger Dryas at Lago Verdarolo ($\sim$ 12,650–11,750 cal BP; Samartin and others 2017) caused notable retreat of mixed deciduous-fir woodlands and re-expansion of steppe-tundra or alpine vegetation (Figure 2). Interestingly, the vegetation around Lago Verdarolo featured a much stronger response to the Younger Dryas cooling than other high-elevation sites on the Northern Apennines, where only minor expansions of steppic plants (for example, *Artemisia*, Chenopodiaceae) and marked reductions of temperate trees occurred (Lowe 1992; Ponel and Lowe 1992; Vescovi and others 2010a).

Abrupt warming at the onset of the Holocene (+ 5 °C from $\sim$ 11,700 to 10,000 cal BP; Samartin and others 2017) triggered the rapid upward expansion of mixed deciduous forests (deciduous *Quercus*, *Ulmus*, *Fraxinus excelsior*, *Betula*, *Corylus*) around Lago Verdarolo, replacing steppic or alpine vegetation and cold-tolerant pinewoods (Figures 2, 3, S2). *Abies alba* expanded slightly later (at $\sim$ 11,000 cal BP), partially outcompeting more light-demanding and shorter trees such as deciduous *Quercus* and *Betula* (Figures 2, S2). Closed mixed forests with *Abies alba* and broadleaved deciduous trees (*Quercus*, *Ulmus*, *Tilia*, *Fraxinus excelsior*) dominated for around 5000 years ($\sim$ 10,500—5400 cal BP) under rather warm ($\sim$ 1—2.5 °C warmer than the mean inferred *T*_July_ for the 2000—100 cal BP period, that is, 1—2 °C warmer than present-day *T*_July_; Figure 2, Samartin and others 2017) and overall dry summer conditions (Magny and others 2007, 2012; Tinner and others 2013). Milligan and others (2020) have recently suggested that this vegetation pattern may have been widespread in central Europe during the Early Holocene.

The multi-millennial persistence of highly diverse mixed forests dominated by *Abies alba* during the Early and Mid-Holocene is a major feature of the vegetation history of the Northern Apennines at high and mid elevations ($\sim$ 800–1800 m asl), also comprising the submediterranean vegetation belt ($\sim$ 200–800 m asl; Lowe 1992; Watson 1996; Cruise and others 2009; Vescovi and others 2010a, b; Branch 2013; Guido and others 2013). On the Mediterranean coast and adjacent mesomediterranean lowlands ($\sim$ 0–200 m asl), *Abies alba* was also co-dominant in the early and Mid Holocene forests, where it coexisted with broadleaved evergreen taxa (for example, *Quercus ilex*, *Arbutus unedo*, *Phillyrea*) in addition to temperate deciduous trees (Colombaroli and others 2007; Bellini and others 2009; Tinner and others 2013). However, the composition and spatial distribution of such mixed forests in the Northern Apennines is still a matter of certain debate. On the one hand, some authors have considered fir-dominated mixed forests with a high diversity of broadleaved deciduous trees as the dominant vegetation at mid-to-high elevations (> 800—1000 m asl; Cruise and others 2009; Vescovi and others 2010a, b; Branch 2013; Guido and others 2013). On the other hand, Watson (1996) proposed the past occurrence of two differentiated forest belts: (1) a lower one consisting in mixed deciduous forests dominated by thermophilous broadleaved trees and (2) an upper one including silver fir forests. Novel macrofossil data from Lago Verdarolo combined with the results of PCA allow refining the aforementioned previous proposals for the mid- and high-elevation forest vegetation of the Northern Apennines during the Early and Mid-Holocene. Combined with the available records our novel evidence suggests that (1) the upper belt ($\sim$ 800–1800 m asl) consisted of mixed forests dominated by *Abies alba* (inferred from the abundance of its macrofossils at Lago Verdarolo; Figure 2) with a diverse array of broadleaved deciduous trees (Figure 3) but probably low abundances of the most thermophilous species (for example, submediterranean *Quercus*), and (2) the lower belt ($\sim$ 200–800 m asl) hosted mixed forests co-dominated by deciduous *Quercus* and *Abies alba* (for example, Cruise 2009; Vescovi and others 2010b; Tinner and others 2013). Further, the Lago Verdarolo record shows that these forests established and flourished under limited fire occurrence (Figure 2), probably resulting from a very moist climate (today’s mean annual precipitation is $\sim$ 2500 mm). Indeed, the results of RDA and GAM suggest the dominant tree species (for example, *Abies alba*, *Ulmus*, *Fraxinus excelsior*, *Tilia*, *Acer*) to have been rather sensitive to fire (Figures 3B, 4, S4), in agreement with previous short-term post-fire monitoring (for example, Delarze and others 1992; Hofmann and others 1998; Thomas 2016; Thomas and others 2018) and long-term paleoecological studies (for example, Tinner and others 1999, 2000; Rey and others 2019).

### Mid-to-Late Holocene Mixed Forest Disruption, Fir Decline and Beech Expansion

The first minor disruption of the mixed fir-dominated forests around 6700 cal BP, involving first forest clearance and the spread of disturbance-tolerant deciduous *Quercus* and *Corylus*, was probably caused by increased fire activity related to pastoral farming (Figure 2). Our interpretation agrees with previous regional paleoecological and archeological evidence on the use of fire by Neolithic settlers to establish pasturelands for their transhumant livestock herds by clearing mixed fir-dominated forests (Vescovi and others 2010a; Branch and Marini 2014). However, the main vegetational shift observed at Lago Verdarolo during the second half of the Holocene was the significant decline of most of the main components of the mixed forests (for example, *Abies alba*, *Fraxinus excelsior*, *Tilia*, *Ulmus*) and the synchronous massive expansion of *Fagus*, starting around 6000 cal BP but particularly striking since about 5400 cal BP (Figure 2). *Fagus sylvatica* was most likely the species of beech involved in this expansion, considering the current distribution of the species (San-Miguel-Ayanz and others 2016) and the available fossil evidence from the Italian Peninsula (Magri 2008). This process lasted remarkably long, coming to an end only about 600 years ago when *Abies alba* went locally extinct. Mid-to-Late Holocene reductions of *Abies alba* and accompanying/co-dominant deciduous trees coupled with expansions of *Fagus sylvatica* were widespread in the montane belt of the Northern Apennines (for example, Lowe 1992; Watson 1996; Vescovi and others 2010a; Branch 2013; Guido and others 2013; Branch and Marini 2014) and also farther south along this mountain range (Allen and others 2002; de Beaulieu and others 2017) as well as north of the Po Plain in the Southern Alps and their forelands (Tinner and others 1999; Gobet and others 2000; Hofstetter and others 2006). As a result, relatively species-poor beech-fir forests replaced the pre-existing highly diverse mixed fir-deciduous forests in the montane belt (Figure 2; Watson 1996; Vescovi and others 2010a). This vegetation shift has been dated in most studied and reasonably well-dated sites between about 7000 and 6000 cal BP in the Northern Apennines (Watson 1996; Vescovi and others 2010a; Branch 2013; Guido and others 2013) and elsewhere in the Italian Peninsula (for example, Tinner and others 1999, 2013; Gobet and others 2000; Hofstetter and others 2006; Colombaroli and others 2007).

Human disturbance was considered the main driver of beech expansion in the Northern Apennines long time ago (see Lowe 1992; Lowe and others 1994), although the precise mechanisms involved remained unclear. Thereafter, and because of insufficient taxonomic resolution of pollen indicators of human impact, Watson (1996) proposed an alternative hypothesis involving climatic forcing as the main driver, assisted by disturbance. Later, Vescovi and others (2010a), based on refined pollen identification and new evidence for fire occurrence derived from quantitative charcoal data, refined the anthropogenic hypothesis, suggesting that human-set fire during the Neolithic promoted the spread of beech by releasing its light-demanding seedlings from the dense shade of fir forest canopy. Finally, Branch (2013) and Branch and Marini (2014) suggested that *Fagus* spread under favorable climatic conditions assisted by human activities such as pastoralism and foddering. However, the lack of pollen-independent climatic reconstructions as well as quantitative records of fire activity hampered testing properly this hypothesis. Here, for the first time, we provide independent paleoclimatic evidence to test these hypotheses. At Lago Verdarolo, mixed species-rich fir-deciduous forests were replaced with *Fagus*-*Abies* forests from around 5400 to 2050 cal BP (Figure 2) under decreasing summer temperatures (− 2 °C, Figure 2; Samartin and others 2017), higher moisture availability (Magny and others 2007, 2012), and slightly increasing farming (continuous curves of *Plantago lanceolata*-t. and *Olea*; Figure 2). Additionally, the charcoal record suggests overall higher fire activity during this period, although evidence is not conclusive (Figure 2). Palynological, paleoclimatic and microclimatic evidence suggests that beech, similarly to silver fir, survived the last glaciation in refugia located in hilly areas of the Po Plain (for example, Colli Euganei) and at the foothills of the Northern Apennines (Magri 2008; Kaltenrieder and others 2009; Samartin and others 2016; Gubler and others 2018; Guido and others 2020) but, in striking contrast to silver fir, beech did not expand until the Mid-Holocene. Our data suggest that the initial expansion of *Fagus sylvatica* occurred when summers got cooler and fire activity rose to moderate values (Figures 2, 4). Likewise, decreasing seasonality and moister summers (Magny and others 2012) may have favored a species very sensitive to late frost such as beech (Packham and others 2012). Additionally, *Fagus sylvatica* is less drought tolerant than *Abies alba*, for instance (Leuschner and Ellenberg 2017). The Lago Verdarolo paleoecological record also shows that the tree species at which expense beech expanded (basically *Abies alba*, *Tilia*, *Ulmus* and *Fraxinus excelsior*) responded quite negatively to decreasing summer temperatures and increasing fire occurrence (Figures 3, 4). Our results mostly agree with previous research on short- and long-term fire ecology of these species: *Fagus sylvatica* is sensitive to fire but given that competing species (*Abies, Ulmus, Tilia, Fraxinus, Picea*) are even more damaged by burning, beech may benefit from moderate-severity fires (Tinner and others 2000; van Gils and others 2010; Packham and others 2012; Ascoli and others 2015; Feurdean and others 2017; Carter and others 2018; Rey and others 2019). We therefore assume that beech-fir forests replaced mixed fir-deciduous stands because beech became more competitive with the onset of cooler and moister summers and human fire disturbance. A similar conclusion was reached by other authors elsewhere in Europe regarding the removal of particularly sensitive tree species (Bradshaw and Lindbladh 2005; Muñoz Sobrino and others 2009). Fir persisted as an important forest component longer than at other sites in the Italian Peninsula because human impact was moderate (Figure 2).

In short, the Lago Verdarolo multi-proxy and high-quality record has allowed us to check the previously formulated competing hypotheses, showing (for the first time with pollen-independent local paleoclimatic reconstructions) that both human disturbance and climate change probably contributed to the Mid-to-Late Holocene expansion of *Fagus sylvatica* in the Northern Apennine forests. Browsing may have also played a major role in favoring *Fagus* against co-existing species more palatable and sensitive to browsing such as *Tilia*, *Ulmus*, *Fraxinus* and *Abies* as proposed by Branch and Marini (2014) but the lack of a proxy for grazing, such as coprophilous fungal spores, prevented us from testing this mechanism at Lago Verdarolo.

### Legacy of Past Human Activities on Modern Forest Composition

The final demise of *Abies alba* at Lago Verdarolo starting around 2500 cal BP was likely triggered by enhanced fire occurrence related to farming activities (Figures 2, 3) and resulted in the dominance of *Fagus sylvatica* in the montane forests, as indicated by pollen and macrofossil data (Figure 2). During the last 2000 years, beech continued its expansion under increasing land-use including arboriculture (*Castanea*, *Juglans*, *Olea*), agriculture (Cerealia-t., *Cannabis*) and pastoralism, which likely involved an intense use of fire (Figure 2). *Abies alba* kept declining in parallel to enhanced human impact and fire occurrence, until it was probably locally extirpated between about 1000 and 500 cal BP according to the macrofossil and pollen records (Figure 2). Similarly, other major trees of the formerly dominant mixed forests such as *Acer*, *Carpinus betulus*, *Fraxinus excelsior*, *Tilia* and *Ulmus* turned extremely rare and probably underwent local extinction (Figure 2). Therefore, the paleoecological data from Lago Verdarolo strongly suggest that the currently widespread (quasi)-monospecific *Fagus sylvatica* stands of the Northern Apennine montane belt are the outcome of millennia of land-use intensification. Beech took advantage of its lower palatability and sensitivity to fire and browsing compared to other co-existing trees to increase its dominance (Figures 3, 4; Pigott 1991; Tinner and others 2000; Thomas 2016; Leuschner and Ellenberg 2017; Thomas and others 2018; Rey and others 2019). Previous paleoecological and archeological studies in the region suggested that ancient farming favored beech directly because of the use of beechnuts to feed pigs and indirectly by overexploiting competing trees like *Ulmus*, *Tilia*, *Fraxinus* and *Abies* for foddering (Cruise and others 2009; Branch 2013; Branch and others 2014). Later, the dense shade of beech stands would have severely hampered seedling recruitment of more light-demanding species (Vescovi and others 2010a; Branch 2013; Leuschner and Ellenberg 2017).

A few beech-fir forests have persisted until the present-day in the Apennines but, with few exceptions, *Abies alba* plays a markedly subordinate role (Watson 1996; Vescovi and others 2010a). Tinner and others (2013) already highlighted that land-use and excessive human-induced fires and pastoralism caused widespread declines of *Abies alba* forests throughout the Italian Peninsula, which has a strong impact on modern forest composition and dynamics. Our results show that the legacy of long-lasting human activities extends to the rarity or even absence of many other mesophilous broadleaved deciduous trees that were relatively frequent in the Early and Mid-Holocene forests. Likewise, the widespread monospecific *Fagus* forests that dominate in the montane belt of the Apennines today have been favored, directly or indirectly, by human activities, as it was the case elsewhere in Europe where the species is quite widespread today (Birks and Tinner 2016).

## Conclusions

In this study, we have shown that highly diverse mixed forests dominated by *Abies alba* and several broadleaved deciduous tree species (for example, *Ulmus*, *Tilia*, *Fraxinus excelsior*, *Acer*) dominated in the Northern Apennines during the Early and Mid-Holocene under warmer (*T*_July_ 1—2 °C higher than today’s climate reference period) and drier summers. These conditions are like those forecasted for the near future under the ongoing climatic change (Kovats and others 2014), so reviving these ancient Early and Mid-Holocene forests could be a feasible possibility to adapt forests to future conditions (Henne and others 2015). This holds particularly true when considering that *Fagus sylvatica*, the currently dominant tree in the montane belt of the Apennines, is way more sensitive to summer drought than most of the tree species dominating during the warmest period of the Holocene (for example, *Abies alba, Fraxinus excelsior, Acer, Tilia, Ulmus*; Pigott 1991; Packham and others 2012; Thomas 2016; Leuschner and Ellenberg 2017; Thomas and others 2018) and may probably experience increasingly frequent and severe diebacks related to drought (Piovesan and others 2008; Dorado-Liñán and others 2019). However, future forest management must also consider that in many locations most of the natural components of the Early and Mid-Holocene mixed forests are currently rare or absent after millennia of intense land-use. Moreover, the moderate-to-high sensitivity to fire of most of these trees might be a major issue that forest management should account for, given that burning may increase in the near future under global warming conditions (Moriondo and others 2006).

## Electronic supplementary material

Below is the link to the electronic supplementary material.

Supplementary material 1 (PDF 1757 kb)

Supplementary material 2 (DOCX 13 kb)

## References

[CR1] Allen JRM, Watts WA, McGee E, Huntley B (2002). Holocene environmental variability–the record from Lago Grande di Monticchio, Italy. Quaternary International.

[CR2] Ascoli D, Vacchiano G, Maringer J, Bovio G, Conedera M (2015). The synchronicity of masting and intermediate severity fire effects favors beech recruitment. Forest Ecology and Management.

[CR3] Bellini C, Mariotti-Lippi M, Montanari C (2009). The Holocene landscape history of the NW Italian coasts. The Holocene.

[CR4] Bennett KD (1996). Determination of the number of zones in a biostratigraphical sequence. New Phytologist.

[CR5] Beug H-J (2004). Leiftaden der Pollenbestimmung für Mitteleuropa und angrezende Gebiete.

[CR6] Birks HJB, Gordon AD (1985). Numerical methods in Quaternary pollen analysis.

[CR7] Birks HH, Elias SA, Mock CJ (2013). Plant Macrofossils: Introduction. Encyclopedia of Quaternary Science.

[CR8] Birks HJB, Tinner W, San-Miguel-Ayanz J, de Rigo D, Caudullo G, Houston Durrant T, Mauri A (2016). Past forests of Europe. European Atlas of Forest Tree Species.

[CR9] Bradshaw RHW, Lindbladh M (2005). Regional spread and stand-scale establishment of *Fagus sylvatica* and *Picea abies* in Scandinavia. Ecology.

[CR10] Branch NP (2013). Early-Middle Holocene vegetation history, climate change and human activities at Lago Riane (Ligurian Apennines, NW Italy). Vegetation History and Archaeobotany.

[CR11] Branch NP, Marini NAF (2014). Mid-Late Holocene environmental change and human activities in the northern Apennines, Italy. Quaternary International.

[CR12] Branch NP, Black S, Maggi R, Marini NAF (2014). The Neolithisation of Liguria (NW Italy): An environmental archaeological and palaeoenvironmental perspective. Environmental Archaeology.

[CR13] Caudullo G, Tinner W, San-Miguel-Ayanz J, de Rigo D, Caudullo G, Houston Durrant T, Mauri A (2016). Abies – Circum-Mediterranean firs in Europe: distribution, habitat, usage and threats. European Atlas of Forest Tree Species.

[CR14] Carter VA, Moravcová A, Chiverrell RC, Clear JL, Finsinger W, Dreslerová D, Halsall K, Kuneš P (2018). Holocene-scale fire dynamics of central European temperate spruce-beech forests. Quaternary Science Reviews.

[CR15] Colombaroli D, Marchetto A, Tinner W (2007). Long-term interactions between Mediterranean climate, vegetation and fire regime at Lago di Massaciuccoli (Tuscany, Italy). Journal of Ecology.

[CR16] Colombaroli D, Henne PD, Kaltenrieder P, Gobet E, Tinner W (2010). Species responses to fire, climate and human impact at tree line in the Alps as evidenced by palaeo-environmental records and a dynamic simulation model. Journal of Ecology.

[CR17] Cruise GM, Macphail RI, Linderholm J, Maggi R, Marshall PD (2009). Lago di Bargone, Liguria, N Italy: a reconstruction of Holocene environmental and land-use history. The Holocene.

[CR18] de Beaulieu J-L, Brugiapaglia E, Joannin S, Guiter F, Zanchetta G, Wulf S, Peyron O, Bernardo L, Didier J, Stock A, Rius D, Magny M (2017). Lateglacial-Holocene abrupt vegetation changes at Lago Trifoglietti in Calabria, Southern Italy: The setting of ecosystems in a refugial zone. Quaternary Science Reviews.

[CR19] Delarze R, Caldelari D, Hainard P (1992). Effects of fire on forest dynamics in southern Switzerland. Journal of Vegetation Science.

[CR20] Dorado-Liñán I, Piovesan G, Martínez-Sancho E, Gea-Izquierdo G, Zang C, Cañellas I, Castagneri D, Di Filippo A, Gutiérrez E, Ewald J, Fernández-de-Uña L, Hornstein D, Jantsch MC, Levanič T, Mellert KH, Vacchiano G, Zlatanov T, Menzel A (2019). Geographical adaptation prevails over species-specific determinism in trees’ vulnerability to climate change at Mediterranean rear-edge forests. Global Change Biology.

[CR21] Feurdean A, Florescu G, Vannière B, Tanţău I, O’Hara RB, Pfeiffer M, Hutchinson SM, Gałka M, Moskal-del Hoyo M, Hickler T (2017). Fire has been an important driver of forest dynamics in the Carpathian Mountains during the Holocene. Forest Ecology and Management.

[CR22] Finsinger W, Tinner W (2005). Minimum count sums for charcoal-concentration estimates in pollen slides: accuracy and potential errors. The Holocene.

[CR23] Finsinger W, Schwörer C, Heiri O, Morales-Molino C, Ribolini A, Giesecke T, Haas JN, Kaltenrieder P, Magyari EK, Ravazzi C, Rubiales JM, Tinner W (2019). Fire on ire and frozen trees? Inappropriate radiocarbon dating leads to unrealistic reconstructions. New Phytologist.

[CR24] Galiano L, Martínez-Vilalta J, Lloret F (2010). Drought-induced multifactor decline of Scots pine in the Pyrenees and potential vegetation change by the expansion of co-occurring oak species. Ecosystems.

[CR25] García-Valdés R, Svenning J-C, Zavala MA, Purves DW, Araújo MB (2015). Evaluating the combined effects of climate and land-use change on tree species distributions. Journal of Applied Ecology.

[CR26] Gazol A, Camarero JJ, Gutiérrez E, Popa I, Andreu-Hayles L, Motta R, Nola P, Ribas M, Sangüesa-Barreda G, Urbinati C, Carrer M (2015). Distinct effects of climate warming on populations of silver fir (*Abies alba*) across Europe. Journal of Biogeography.

[CR27] Gobet E, Tinner W, Hubschmid P, Jansen I, Wehrli M, Ammann B, Wick L (2000). Influence of human impact and bedrock differences on the vegetational history of the Insubrian Southern Alps. Vegetation History and Archaeobotany.

[CR28] Gubler M, Henne PD, Schwörer C, Boltshauser-Kaltenrieder P, Lotter AF, Brönnimann S, Tinner W (2018). Microclimatic gradients provide evidence for a glacial refugium for temperate trees in a sheltered hilly landscape of Northern Italy. Journal of Biogeography.

[CR29] Guido MA, Menozzi BI, Bellini C, Placereani S, Montanari C (2013). A palynological contribution to the environmental archaeology of a Mediterranean mountain wetland (North West Apennines, Italy). The Holocene.

[CR30] Guido MA, Molinari C, Moneta V, Branch N, Black S, Simmonds M, Stastney P, Montanari C (2020). Climate and vegetation dynamics of the Northern Apennines (Italy) during the Late Pleistocene and Holocene. Quaternary Science Reviews.

[CR31] Hastie TJ, Tibshirani RJ (1990). Generalized Additive Models.

[CR32] Heegard E, Birks HJB, Telford RJ (2005). Relationships between calibrated ages and depth in stratigraphical sequences: an estimation procedure by mixed-effect regression. The Holocene.

[CR33] Henne PD, Elkin C, Franke J, Colombaroli D, Calò C, La Mantia T, Pasta S, Conedera M, Dermody O, Tinner W (2015). Reviving extinct Mediterranean forest communities may improve ecosystem potential in a warmer future. Frontiers in Ecology and the Environment.

[CR34] Hofmann C, Conedera M, Delarze R, Carraro G, Giorgetti P (1998). Effets des Incendies de forêt sur la végétation au Sud des Alpes suisses. Mitteilungen der Eidgenössischen Forschungsanstalt für Wald, Schnee und Landschaft.

[CR35] Hofstetter S, Tinner W, Valsecchi V, Carraro G, Conedera M (2006). Lateglacial and Holocene vegetation history in the Insubrian Southern Alps—New indications from a small-scale site. Vegetation History and Archaeobotany.

[CR36] Kaltenrieder P, Belis CA, Hofstetter S, Ammann B, Ravazzi C, Tinner W (2009). Environmental and climatic conditions at a potential glacial refugial site of tree species near the Southern Alpine glaciers. New insights from multiproxy sedimentary studies at Lago della Costa (Euganean Hills, Northeastern Italy). Quaternary Science Reviews.

[CR37] Kovats RS, Valentini R, Bouwer LM, Georgopoulou E, Jacob D, Martin E, Rounsevell M, Soussana J-F, Barros VR, Field CB, Dokken DJ, Mastrandrea MD, Mach KJ, Bilir TE, Chatterjee M, Ebi KL, Estrada YO, Genova RC, Girma B, Kissel ES, Levy AN, MacCracken S, Mastrandrea PR, White LL (2014). Europe. Climate Change 2014: Impacts, Adaptation, and Vulnerability. Part B: Regional Aspects. Contribution of Working Group II to the Fifth Assessment Report of the Intergovernmental Panel on Climate Change.

[CR38] Legendre P, Birks HJB, Birks HJB, Lotter AF, Juggins S, Smol JP (2012). From classical to canonical ordination. Tracking Environmental Change Using Lake Sediments. Data Handling and Numerical Techniques 5.

[CR39] Leuschner C, Ellenberg H (2017). Ecology of Central European Forests Vegetation and Ecology of Central Europe.

[CR40] Lévesque PEM. 1998. Guide to the identification of plant macrofossils in Canadian peatlands. Publication No. 1817. Montreal (Canada): Agriculture Canada.

[CR41] Lowe JJ (1992). Lateglacial and early Holocene lake sediments from the northern Apennines, Italy—pollen stratigraphy and radiocarbon dating. Boreas.

[CR42] Lowe JJ, Davite C, Moreno D, Maggi R (1994). Holocene pollen stratigraphy and human interference in the woodlands of the northern Apennines, Italy. The Holocene.

[CR43] Magny M, de Beaulieu J-L, Drescher-Schneider R, Vannière B, Walter-Simonnet A-V, Millet L, Bossuet G, Peyron O (2006). Climatic oscillations in central Italy during the Last Glacial-Holocene transition: the record from Lake Accesa. Journal of Quaternary Science.

[CR44] Magny M, de Beaulieu J-L, Drescher-Schneider R, Vannière B, Walter-Simonnet A-V, Miras Y, Millet L, Bossuet G, Peyron O, Brugiapaglia E, Leroux A (2007). Holocene climate changes in the central Mediterranean as recorded by lake-level fluctuations at Lake Accesa (Tuscany, Italy). Quaternary Science Reviews.

[CR45] Magny M, Peyron O, Sadori L, Ortu E, Zanchetta G, Vannière B, Tinner W (2012). Contrasting patterns of precipitation seasonality during the Holocene in the south- and north-central Mediterranean. Journal of Quaternary Science.

[CR46] Magri D (2008). Patterns of post-glacial spread and the extent of glacial refugia of European beech (*Fagus sylvatica*). Journal of Biogeography.

[CR47] Milligan G, Bradshaw RHW, Clancy D, Zychaluk K, Spencer M (2020). Effects of human land use and temperature on community dynamics in European forests. Quaternary Science Reviews.

[CR48] Moore PD, Webb JA, Collinson ME (1991). Pollen analysis.

[CR49] Moriondo M, Good P, Durao R, Bindi M, Giannakopoulos C, Corte-Real J (2006). Potential impact of climate change on fire risk in the Mediterranean area. Climate Research.

[CR50] Muñoz Sobrino C, Ramil-Rego P, Gómez-Orellana L, Ferreiro da Costa J, Díaz Varela RA (2009). Climatic and human effects on the post-glacial dynamics of Fagus sylvatica L. in NW Iberia. Plant Ecology.

[CR51] Packham JR, Thomas PA, Atkinson MD, Degen T (2012). Biological Flora of the British Isles: *Fagus sylvatica*. Journal of Ecology.

[CR52] Pigott CD (1991). Biological Flora of the British Isles: *Tilia cordata*. Journal of Ecology.

[CR53] Piovesan G, Biondi F, Di Filippo A, Alessandrini A, Maugeri M (2008). Drought-driven growth reduction in old beech (*Fagus sylvatica* L.) forests of the central Apennines. Italy. Global Change Biology.

[CR54] Ponel P, Lowe JJ (1992). Coleopteran, pollen and radiocarbon evidence from the Prato Spilla “D” succession, N Italy. Comptes Rendus Academie Science Paris.

[CR55] Reille M (1992). Pollen et spores d’Europe et d’Afrique du Nord.

[CR56] Reimer PJ (2013). IntCal13 and Marine13 radiocarbon age calibration curves 0–50,000 years cal BP. Radiocarbon.

[CR57] Rey F, Gobet E, Schwörer C, Wey O, Hafner A, Tinner W (2019). Causes and mechanisms of synchronous succession trajectories in primeval Central European mixed *Fagus sylvatica* forests. Journal of Ecology.

[CR58] Ruiz-Benito P, Ratcliffe S, Zavala MA, Martínez-Vilalta J, Vilà-Cabrera A, Lloret F, Madrigal-González J, Wirth C, Greenwood S, Kändler G, Lehtonen A, Kattge J, Dahlgren J, Jump AS (2017). Climate- and successional-related changes in functional composition of European forests are strongly driven by tree mortality. Global Change Biology.

[CR59] Sabatini FM, Burrascano S, Keeton WS, Levers C, Lindner M, Pötzschner F, Verkerk PJ, Bauhus J, Buchwald E, Chaskovsky O, Debaive N, Horváth F, Garbarino M, Grigoriadis N, Lombardi F, Marques Duarte I, Meyer P, Midteng R, Mikac S, Mikoláš M, Motta R, Mozgeris G, Nunes L, Panayotov M, Ódor P, Ruete A, Simovski B, Stillhard J, Svoboda M, Szwagrzyk J, Tikkanen O-P, Volosyanchuk R, Vrska T, Zlatanov T, Kuenmerle T (2018). Where are Europe’s last primary forests?. Diversity and Distributions.

[CR60] Samartin S, Heiri O, Lotter AF, Tinner W (2012). Climate warming and vegetation response after Heinrich event 1 (16 700–16 000 cal yr BP) in Europe south of the Alps. Climate of the Past.

[CR61] Samartin S, Heiri O, Vescovi E, Brooks SJ, Tinner W (2012). Lateglacial and early Holocene summer temperatures in the southern Swiss Alps reconstructed using fossil chironomids. Journal of Quaternary Science.

[CR62] Samartin S, Heiri O, Kaltenrieder P, Kühl N, Tinner W (2016). Reconstruction of full glacial environments and summer temperatures from Lago della Costa, a refugial site in Northern Italy. Quaternary Science Reviews.

[CR63] Samartin S, Heiri O, Joos F, Renssen H, Franke J, Brönnimann S, Tinner W (2017). Warm Mediterranean mid-Holocene summers inferred from fossil midge assemblages. Nature Geoscience.

[CR64] San-Miguel-Ayanz J, de Rigo D, Caudullo G, Houston Durrant T, Mauri A (2016). European Atlas of Forest Tree Species.

[CR65] Schoch WH, Pawlik B, Schweingruber PH (1988). Botanische Makroreste: ein Atlas zur Bestimmung häufig gefundener und ökologisch wichtiger Pflanzensamen.

[CR66] Šmilauer P, Lepš J (2014). Multivariate analysis of ecological data using Canoco 5.

[CR67] Stockmarr J (1971). Tablets with spores used in absolute pollen analysis. Pollen et spores.

[CR68] ter Braak CJF, Šmilauer P. 2012. Canoco reference manual and user’s guide: software for ordination, version 5.0. Ithaca (NY, USA): Microcomputer Power.

[CR69] Thomas PA (2016). Biological Flora of the British Isles: *Fraxinus excelsior*. Journal of Ecology.

[CR70] Thomas PA, Stone D, La Porta N (2018). Biological Flora of the British Isles: *Ulmus glabra*. Journal of Ecology.

[CR71] Thuiller W, Lavorel S, Araújo MB, Sykes MT, Prentice IC (2005). Climate change threats to plant diversity in Europe. Proceedings of the National Academy of Sciences.

[CR72] Tinner W, Hubschmid P, Wehrli M, Ammann B, Conedera M (1999). Long-term forest fire ecology and dynamics in southern Switzerland. Journal of Ecology.

[CR73] Tinner W, Conedera M, Gobet E, Hubschmid P, Wehrli M, Ammann B (2000). A palaeoecological attempt to classify fire sensitivity of trees in the southern Alps. The Holocene.

[CR74] Tinner W, Hu FS (2003). Size parameters, size-class distribution and area-number relationship of microscopic charcoal: relevance for fire reconstruction. The Holocene.

[CR75] Tinner W, Colombaroli D, Heiri O, Henne PD, Steinacher M, Untenecker J, Vescovi E, Allen JRM, Carraro G, Conedera M, Joos F, Lotter AF, Luterbacher J, Samartin S, Valsecchi V (2013). The past ecology of *Abies alba* provides new perspectives on future responses of silver fir forests to global warming. Ecological Monographs.

[CR76] Tomlinson P (1995). An aid to the identification of fossil buds, bud-scales and catkin-bracts of British trees and shrubs. Circaea.

[CR77] Tzedakis PC, Emerson BC, Hewitt GM (2013). Cryptic or mystic? Glacial tree refugia in northern Europe. Trends in Ecology and Evolution.

[CR78] Vacchiano G, Garbarino M, Lingua E, Motta R (2017). Forest dynamics and disturbance regimes in the Italian Apennines. Forest Ecology and Management.

[CR79] van Gils H, Odoi JO, Andrisano T (2010). From monospecific to mixed forest after fire? An early forecast for the montane belt of Majella, Italy. Forest Ecology and Management.

[CR80] Vescovi E, Ammann B, Ravazzi C, Tinner W (2010). A new Late-glacial and Holocene record of vegetation and fire history from Lago del Greppo, northern Apennines, Italy. Vegetation History and Archaeobotany.

[CR81] Vescovi E, Kaltenrieder P, Tinner W (2010). Late-Glacial and Holocene vegetation history of Pavullo nel Frignano (Northern Apennines, Italy). Review of Palaeobotany and Palynology.

[CR82] Watson CS (1996). The vegetational history of the northern Apennines, Italy: information from three new sequences and a review of regional vegetation change. Journal of Biogeography.

