# Supplementary material for: Long-Term Responses of Mediterranean Mountain Forests to Climate Change, Fire and Human Activities in the Northern Apennines (Italy)
Source: Ecosystems. 2020 Dec 2;24(6):1361–77. doi: 10.1007/s10021-020-00587-4 (PMC7710158; doi:10.1007/s10021-020-00587-4)
Supplement: Supplementary file 1 — Supplementary material 1 (PDF 1757 kb) [file 10021_2020_587_MOESM1_ESM.pdf]

SUPPLEMENTARY INFORMATION

Long-term responses of Mediterranean mountain forests to climate change, fire and human activities in the Northern Apennines (Italy)

César Morales-Molino,[1, 2]* Marianne Steffen,[1] Stéphanie Samartin,[1] Jaqueline F.N. van Leeuwen,[1] Daniel Hürlimann,[1] Elisa Vescovi,[1] and Willy Tinner[1]

[1] Institute of Plant Sciences and Oeschger Centre for Climate Change Research, University of Bern, Altenbergrain 21, CH-3013 Bern, Switzerland

[2] Swiss Federal Institute for Forest, Snow and Landscape Research WSL, A Ramél 18, CH-6593 Cadenazzo, Switzerland

*Corresponding author; e-mail: cesar.morales@ips.unibe.ch

Table S1. Radiocarbon dates from the Lago Verdarolo sedimentary sequence (originally published in Samartin and others 2017).

Laboratory No.	Depth (cm)	Material dated	Radiocarbon date (^{14}C BP)	Calibrated age (cal BP, 95% CI)	Calibrated age (cal BP, median)
Poz-37418	88-90	*F. sylvatica* leaves	75 ± 30	27—259	103
Poz-32844	208-210	*F. sylvatica* leaves, *A. alba* needles	635 ± 30	553—665	599
Poz-37420	288-290	*F. sylvatica* leaves, Wood	1510 ± 30	1327—1520	1392
Poz-39388	319	Wood, periderm	1825 ± 30	1634—1862	1765
Poz-32845	368-370	*A. alba* needles, *F. sylvatica* leaves	2420 ± 30	2351—2696	2446
Poz-39389	410-412	*F. sylvatica* leaves, Wood	3020 ± 30	3080—3340	3215
Poz-37421	449-451	*A. alba* needles, *F. sylvatica* leaves	2405 ± 35*	*Rejected*	*Rejected*
Poz-39390	530-531	Wood	4450 ± 30	4965—5286	5123
Poz-32846	568-570	*A. alba* needles, *F. sylvatica* leaves	5090 ± 40	5743—5917	5818
Poz-37422	649-651	*A. alba* needles, *F. sylvatica* leaves	6460 ± 40	7289—7434	7371
Poz-32847	728-730	*A. alba* needles, *F. sylvatica* leaves	8210 ± 50	9019—9394	9175
Poz-39392	760-762	*A. alba* needles, indet. bud	8820 ± 50	9686—10155	9875
Poz-39393	780-782	*F. sylvatica* leaves, indet. twig, wood	9500 ± 50	10589—11080	10785
Poz-37423	829-833	Indet. charred material, wood, bark, indet. leaf fragments	11850 ± 70	13481—13791	13663

F. sylvatica = *Fagus sylvatica*, *A. alba* = *Abies alba*, cal BP = calendar years before present (0 cal BP = 1950 CE). *Date rejected.

Radiocarbon dates calibrated using CALIB 7.1 and the IntCal13 calibration curve (Reimer and others 2013).

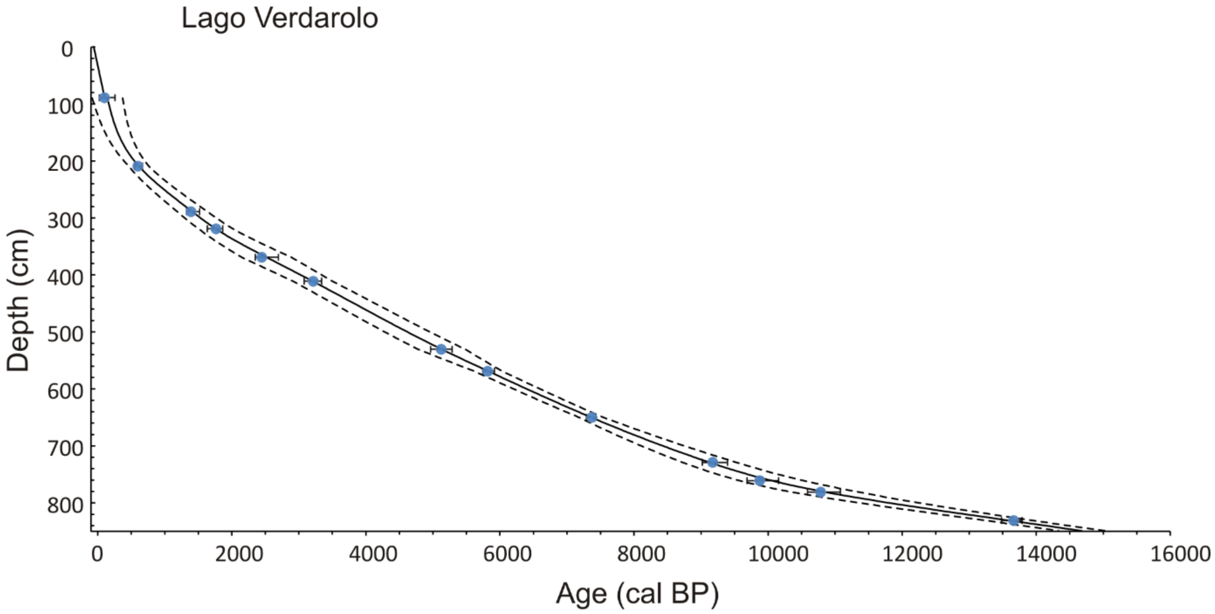

Figure S1. Age-depth model for the sedimentary sequence of Lago Verdarolo obtained using generalized mixed-effect regression within the framework of generalized additive modeling (Heegard and others 2005). Accepted radiocarbon dates (calibrated ages using IntCal13; Reimer and others 2013) denoted by blue dots (error bars delimit the 95% confidence interval of the calibrated ages). The solid line represents the age-depth model; the dashed lines delimit the 95% confidence interval of the modelled ages. Redrawn from Samartin and others (2017). Age expressed in calendar years before present (cal BP; 0 cal BP = 1950 CE).

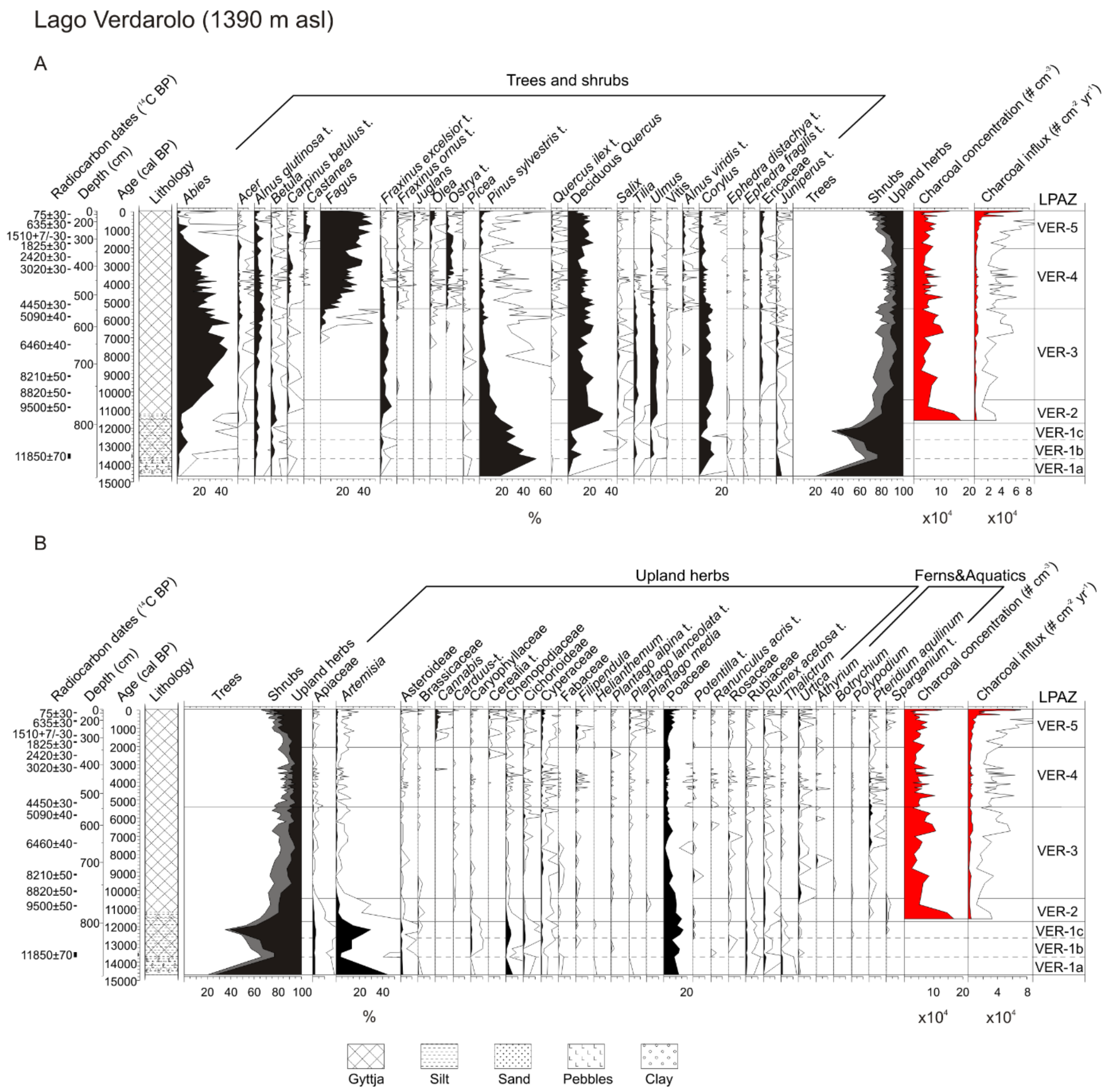

Figure S2. Percentage pollen diagram (selected taxa) and microscopic charcoal data (concentration and influx) from Lago Verdarolo (Northern Apennines, Italy). **(A)** Trees, shrubs and major anthropogenic indicators. **(B)** Upland herbs, ferns and aquatics. Age expressed in calendar years before present (cal BP; 0 cal BP = 1950 CE). Abbreviations: t. = type, LPAZ = Local Pollen Assemblage Zone.

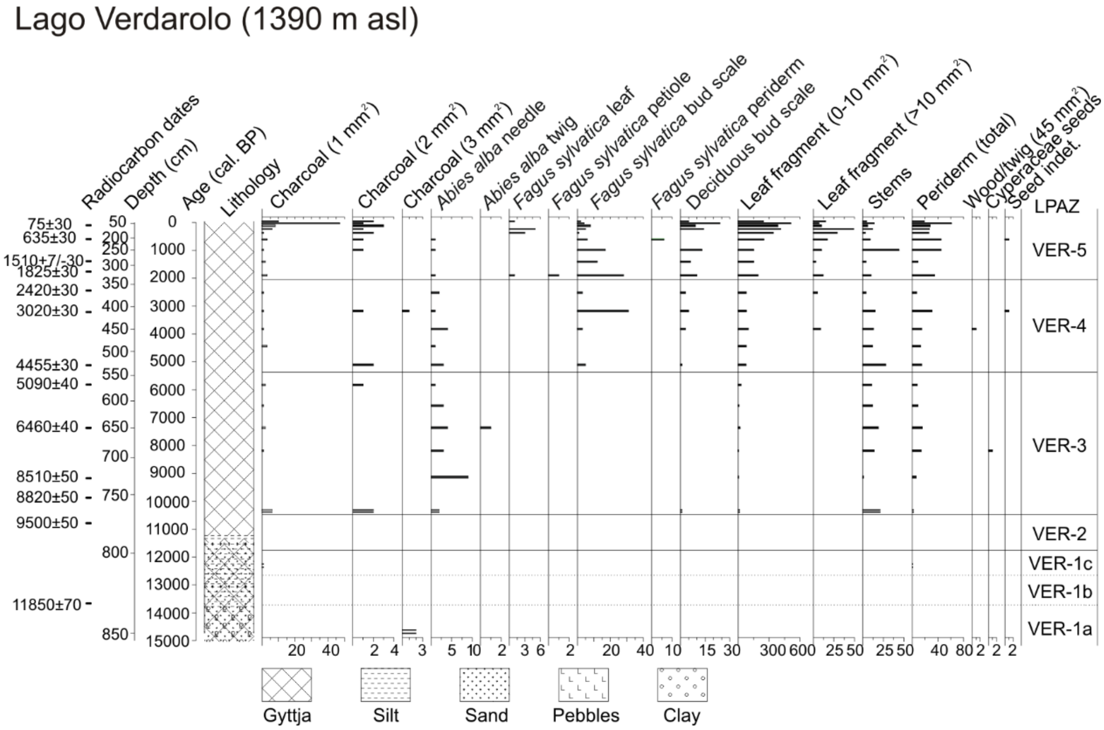

Figure S3. Macrofossil diagram from Lago Verdarolo (Northern Apennines, Italy) in number of remains per 10 cm^3 of sediment. On the left, location of the radiocarbon dates used to establish the chronology of the sequence. Age expressed in calendar years before present (cal BP; 0 cal BP = 1950 CE). LPAZ = Local Pollen Assemblage Zone.

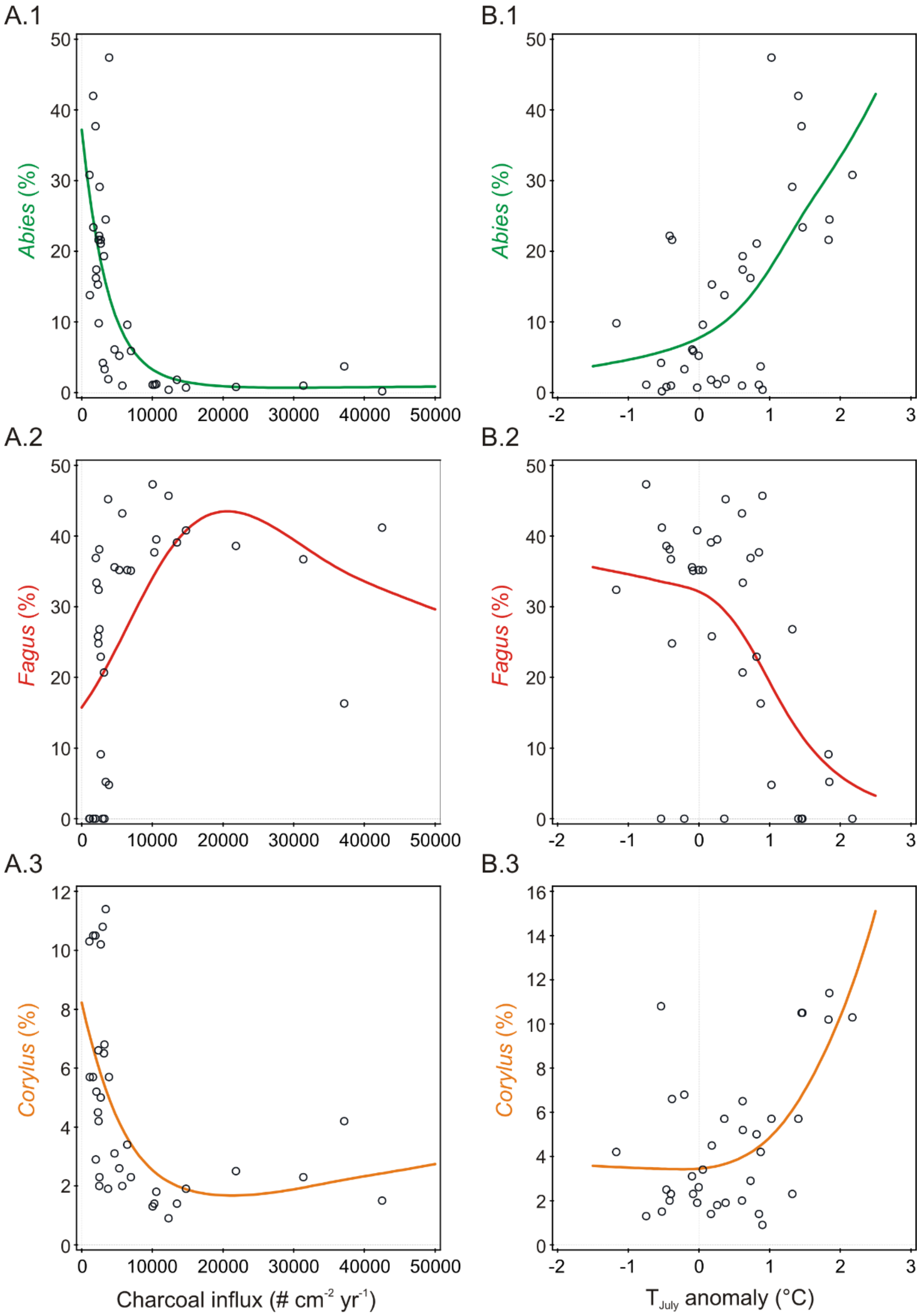

Figure S4. Response curves of the main trees and shrubs (1. *Abies*, 2. *Fagus*, 3. *Corylus*, 4. *Fraxinus excelsior*-t., 5. *Tilia*, 6. *Ulmus*) in the pollen record from Lago Verdarolo to **(A)** fire activity (inferred from microscopic charcoal influx) and **(B)** mean July air temperature anomalies with respect to the mean of the period 2000-100 cal BP (inferred from chironomid assemblages; Samartin and others 2017) fitted using General Additive Models (GAM). Hollow circles represent the samples with pollen, charcoal and chironomid data.

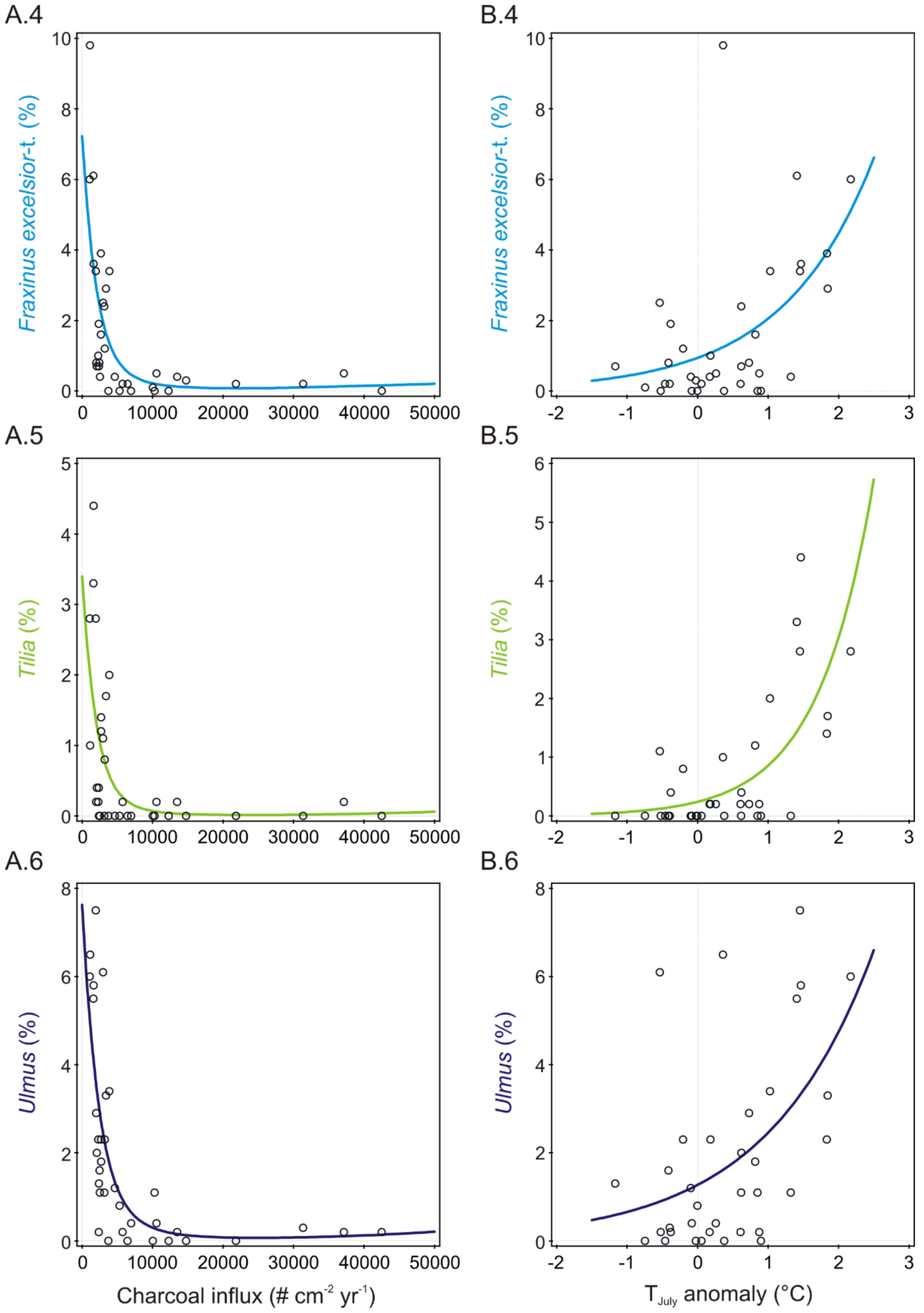

Figure S4 (cont.).

REFERENCES

Heegard E, Birks HJB, Telford RJ. 2005. Relationships between calibrated ages and depths in stratigraphical sequences: an estimated procedure by mixed-effect regression. The Holocene 15: 612-618.

Reimer P and others. 2013. IntCal13 and MARINE13 radiocarbon age calibration curves 0-50000 years cal BP. Radiocarbon 55: 1869-1887.

Samartin S, Heiri O, Joos F, Renssen H, Franke J, Brönnimann S, Tinner W. 2017. Warm Mediterranean mid-Holocene summers inferred from fossil midge assemblages. Nature Geoscience 10: 207-212.
