# Supplementary material for: Long-Term Responses of Mediterranean Mountain Forests to Climate Change, Fire and Human Activities in the Northern Apennines (Italy)
Source: Ecosystems. 2020 Dec 2;24(6):1361–77. doi: 10.1007/s10021-020-00587-4 (PMC7710158; doi:10.1007/s10021-020-00587-4)
Supplement: Supplementary file 2 — Supplementary material 2 (DOCX 13 kb) [file 10021_2020_587_MOESM2_ESM.docx]

ECOSYSTEMS MANUSCRIPT INFORMATION SHEET

MANUSCRIPT NUMBER: ECO-20-0248.R1

TITLE: Long-term responses of Mediterranean mountain forests to climate change, fire and human activities in the Northern Apennines (Italy)

AUTHORS: Morales-Molino, Cesar; Steffen, Marianne; Samartin, Stéphanie; van Leeuwen, Jacqueline; Hürlimann, Daniel; Vescovi, Elisa; Tinner, Willy

CORRESPONDING AUTHOR:

Dr. Cesar Morales-Molino

Institute of Plant Sciences and Oeschger Centre for Climate Change Research University of Bern Altenbergrain 21 Bern Bern 3013 Switzerland

FAX:

PHONE:

EMAIL: [cesarmoralesdelmolino@gmail.com](mailto:cesarmoralesdelmolino@gmail.com), [cesar.morales@ips.unibe.ch](mailto:cesar.morales@ips.unibe.ch)

RECEIVED 30-Jun-2020; ACCEPTED 01-Nov-2020

COLOR FIGURES: 4

COMMENTS:
